# Enhanced neoangiogenesis and balance of the immune response mediated by the Wilms' tumor suppressor WT1 favor repair after myocardial infarction

**DOI:** 10.7150/thno.104329

**Published:** 2025-06-09

**Authors:** Nicole Wagner, Ana Vukolic, Delphine Baudouy, Sophie Pagnotta, Jean-Francois Michiels, Kay-Dietrich Wagner

**Affiliations:** 1 CNRS, INSERM, iBV, Université Côte d'Azur, 06107 Nice, France.; 2 Department of Cardiology, CHU Nice, 06107 Nice, France.; 3 Centre Commun de Microscopie Appliquée, Université Nice Côte d'Azur, Parc Valrose, Nice 06108, France.; 4 Department of Pathology, CHU Nice, 06107 Nice, France.

**Keywords:** myocardial infarction, angiogenesis, immune response, cardiac repair, Wilms' tumor suppressor

## Abstract

**Rationale:** Cardiac repair and regeneration are severely constrained in adult mammals. Several cell types have been identified as playing a role in cardiac repair. However, our understanding of the regulatory proteins common to these cell types and implicated in cardiac repair remains limited.

**Methods:** Experimental myocardial infarctions (MI) were induced in mice by ligation of the left coronary artery. WT1 expression in different cell types was determined by immunofluorescent double-labelling. VE-cadherin-CreERT2 (VE-CreERT2) mice were crossed with Wt1^lox/lox^ animals to generate the VE-CreERT2;Wt1^lox/lox^ strain to knockout WT1 in endothelial cells. Wt1^lox/lox^ and Tie2-CreERT2 animals were crossed to generate Tie2-CreERT2;Wt1^lox/lox^ mice to delete WT1 in endothelial and myeloid-derived cells.

**Results:** We show that the Wilms' tumor suppressor WT1 is expressed in progenitor cell populations, endothelial cells, and myeloid-derived suppressor cells (MDSCs) in mice following MI. Endothelial-specific knockout of WT1 results in reduced vascular density after MI but does not affect functional recovery. Conversely, combined knockout of WT1 in endothelial and myeloid-derived cells increases infarct size, cardiac hypertrophy, fibrosis, hypoxia, and lymphocyte infiltration. Notably, angiogenesis, infiltration of MDSCs, and cellular proliferation were diminished, and importantly, cardiac function was reduced. Mechanistically, in addition to the previously established role of WT1 in promoting the expression of angiogenic molecules, this transcription factor positively regulates the expression of Cd11b and Ly6G, which are crucial for MDSC invasion, migration and function thereby preventing overactivation of the immune response.

**Conclusions:** Several molecules have been identified that are implicated in distinct aspects of cardiac repair following MI. The identification of WT1 as a transcription factor that is essential for repair mechanisms involving various cell types within the heart may potentially enable the future development of a coordinated repair process following myocardial infarction.

## Introduction

According to World Health Organization data, ischemic heart disease is the most common cause of death with the largest increase in the number of cases over the last two decades worldwide (https://www.who.int/news-room/fact-sheets/detail/the-top-10-causes-of-death). Ischemic heart disease could cause myocardial infarction and heart failure 1. Conventional pharmacological therapies of heart failure after myocardial infarction (MI) reduce overall mortality but are non-curative as the regeneration potential of adult mammalian hearts is limited 2. In contrast, fish, amphibians, and neonatal mice are able to regenerate a damaged heart 3-5 (reviewed in 6). Cardiac regeneration is a finely tuned process involving neo-angiogenesis, immune response, epicardial activation, matrix remodeling, progenitor cell activation and cardiomyocyte proliferation 2. Although these processes are frequently investigated separately in cardiac regeneration, the different cell types influence each other, e.g. endothelial cell-derived factors promote cardiomyocyte survival, cardiomyocyte-derived factors induce angiogenesis, immune cells might stimulate neoangiogenesis and cardiomyocyte survival, and epicardial-derived cells might contribute to endothelial cells and cardiomyocytes after myocardial infarction 2,7-10. Thus, identification of common factors in the different cell types, which contribute to cardiac regeneration might offer the possibility to improve cardiac repair in the future. Even in this case, the temporal and spatial control of potential common regulators remains an important issue.

Regeneration recapitulates to some extend the genetic programs of organ formation during embryonic development 2. The Wilms' tumor suppressor WT1 is an important transcriptional regulator of cardiac development. As a transcription factor it can either activate or repress various target genes. Thus, WT1 influences cellular growth, apoptosis, differentiation, and metabolism. WT1 exists in multiple isoforms. Alternative splicing of exon 5 and exon 9 gives rise to major isoforms. Splicing of exon 9 generates the KTS isoforms, which either include or exclude three amino acids lysin, threonine, and serin (KTS) between zinc fingers 3 and 4 of the protein 11. Wt1 knockout mice showed besides kidney and gonad agenesis and various developmental defects a severe cardiac hypoplasia and die during mid-gestation 12-20. Also in humans, mutations in the WT1 gene have been associated with cardiac malformations 21,22 and with cardiac hypertrophy and death due to myocardial infarction in an infant 23. WT1 is expressed in a proportion of epicardial cells; whether these cells contribute to neoangiogenesis or cardiomyocyte generation in repair is a matter of debate 24-29. Developmental expression of WT1 in endothelial cells is required for cardiac angiogenesis and myocardial development 15,20. Furthermore, WT1 is re-expressed in coronary endothelium and vascular smooth muscle cells 12,30, in cardiomyocytes 12,31, and fibroblasts 32 after myocardial infarction in mice and in addition in a pro-regenerative population of macrophages in zebrafish 33. WT1 is required for developmental cardiac vessel formation via activation of the TrkB neurotrophin receptor in mice 15. Also, brain derived neurotrophic factor (BDNF), the ligand for TrkB is an endothelial cell survival factor required for intramyocardial vessel stabilization 34. In addition, we showed earlier that WT1 is involved in endothelial cell proliferation and migration 35, which was confirmed recently 36. Besides the BDNF/TrkB system, WT1 regulates multiple angiogenic molecules e.g. VEGF 37,38, Ets-1 35, nestin 39, nephrin 40,41, TRF2 42, CD31, and c-kit (CD117) 43 in different cellular systems. WT1 is involved in cardiomyocyte differentiation and prevents cardiomyocyte damage in neonatal cardiomyocytes *in vitro* and in adult hearts *in vivo* 12,31. In tumors, WT1 is expressed in mouse and human myeloid-derived suppressor cells (MDSCs), where it is required to suppress T cell proliferation 43. We show here that following MI, WT1 is expressed in progenitor cell populations, endothelial cells, and MDSCs in mice. By generating two inducible knockout models, we provide evidence that WT1 is not only required for angiogenesis but also for MDSC function to prevent overactivation of the immune system and consequently functional recovery of the heart after MI.

## Results

### WT1 is re-expressed in several cell types after myocardial infarction

In this study, we conducted a comprehensive analysis of WT1 expression in murine models of experimental myocardial infarction, induced by left coronary artery ligation, in the acute phase (48-72 hours post-MI) and the reparative phase (3 weeks). The temporal control of these two phases is of paramount importance in the study of cardiac repair. Subsequently, we employed inducible knockout mouse models to elucidate the role of WT1 in various cell types at the aforementioned two time points, thereby facilitating our understanding of the spatial and temporal regulation. The subsequent description will address the expression pattern of WT1 following MI, followed by a detailed analysis of the knockout models during the acute phase (48-72 hours post-MI) and the reparative phase (3 weeks).

**Figure 1:**
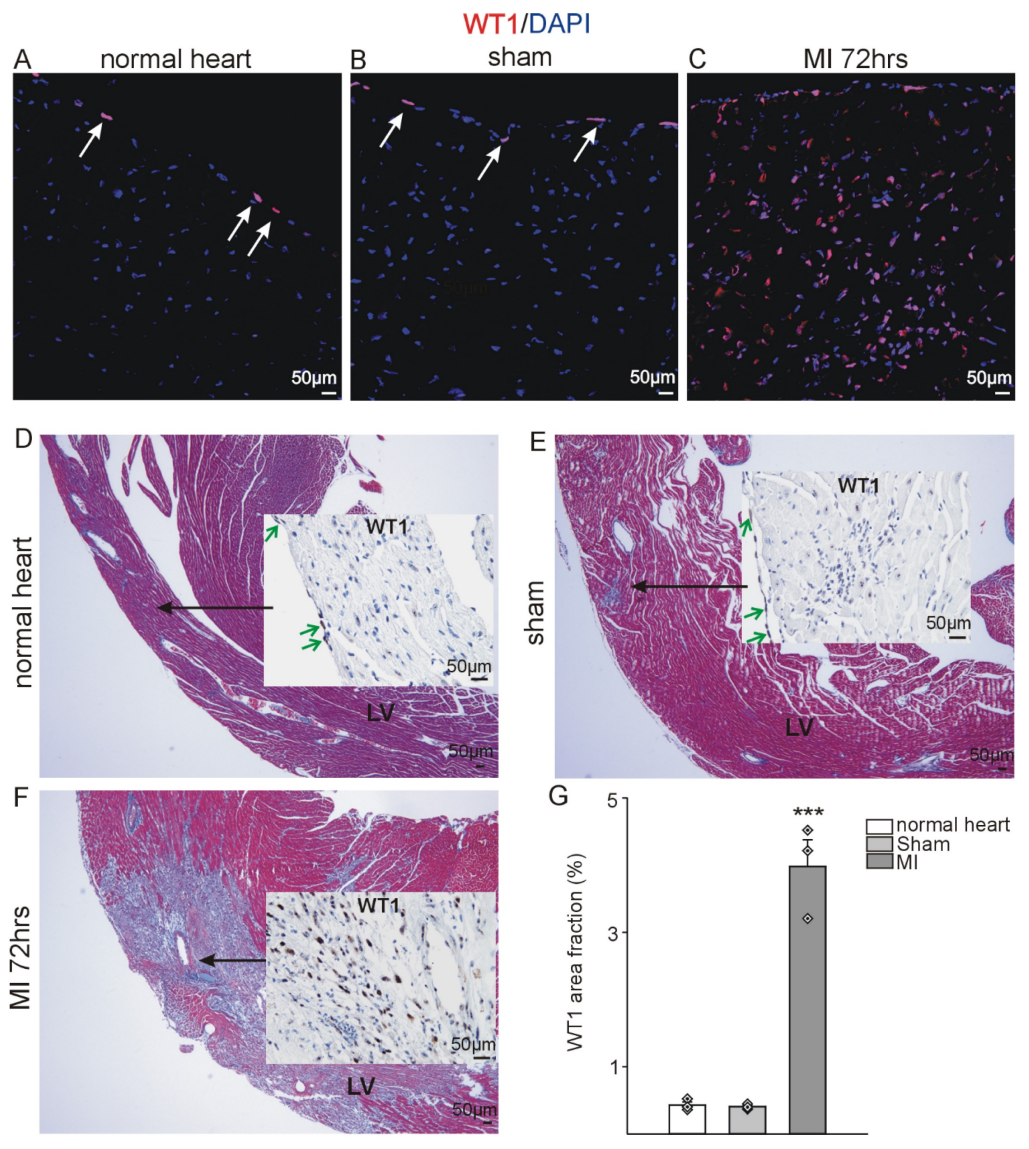
** WT1 is upregulated after myocardial infarction.** Left-ventricular WT1 protein expression (red, DAPI counterstain, blue) in adult (A) healthy mouse hearts, (B) sham-operated and (C) animals 72hrs after MI. Trichrome Masson-stained overviews of the left ventricle (LV) of (D) healthy mouse hearts, (E) sham-operated and (F) animals 72hrs after MI with insertion of representative WT1 immunostainings of this region for subsequent quantification. Arrows (white in (A) and (B), green in (D) and (E)) point to the epicardium where physiological WT1 expression can be seen in controls and sham operated animals, whereas in addition to WT1 expressing epicardial cells, a high number of cells in the left ventricle express WT1 after myocardial infarction. (G) Quantification of WT1 signal area fractions of left ventricle sections from healthy, sham-operated and animals after acute MI (*n =* 3 each). Data are mean ± SEM. ***p ˂ 0.001.

**Figure 2:**
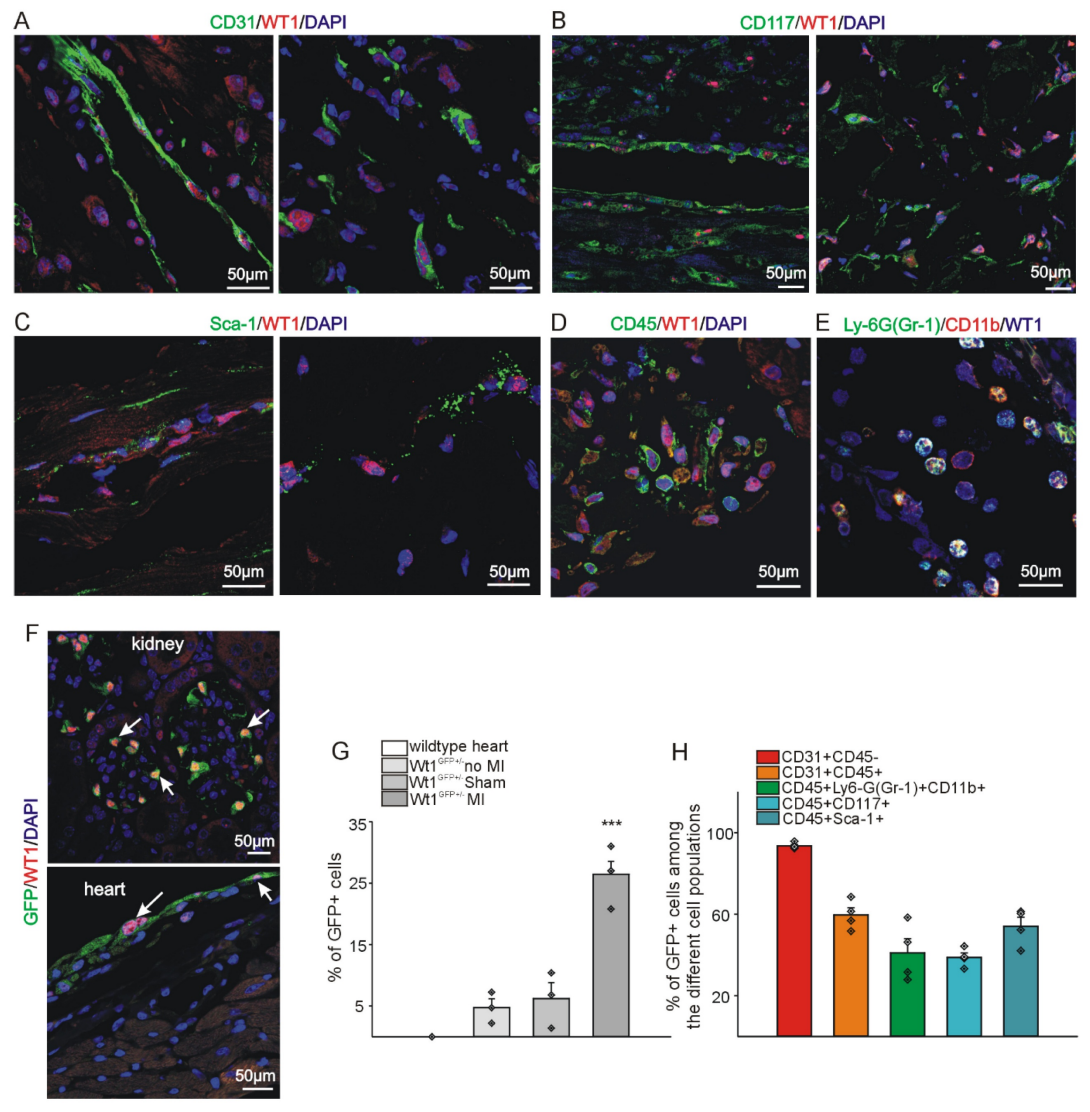
** WT1 is expressed in a high fraction of different cell types after myocardial infarction.** Co-localization of WT1 expression (red) with (A) CD31, (B) CD117, and (C) Sca-1 (green) in vessels and stroma of the left ventricle 72hrs after MI. DAPI (blue) served as counterstain. Co-expression of (D) WT1 (red) and CD45 (green) and (E) WT1 (blue), Ly-6G (Gr-1) (green), and CD11b (red) in cells of the left ventricle 72hrs after MI. (F) GFP (green) and WT1 (red) double-staining of heart and kidney confirms correct co-expression of WT1 and GFP in WT1-GFP knock-in mice. DAPI (blue) served as counterstain in (A,B,C,D,F). White arrows point to some representative kidney podocytes and epicardial cells of the heart. Scale bars indicate 50µm. (G) ImageStream® based quantitative analysis of the percentage of WT1 expressing cells under different conditions using WT1-GFP knock-in mice and wildtype animals. No WT1/GFP expression could be detected in wildtype animals. A low percentage of cells (approx. 5%) expressed WT1/GFP in healthy and sham-operated mouse hearts. In contrast, 72hrs after MI, approx. 25% of cells in the heart were WT1/GFP positive (*n =* 3 each). (h) ImageStream® analysis of the fraction of WT1/GFP expressing cells in different cell populations i.e. endothelial cells (CD31^+^CD45^-^), hematopoietic/myeloid cells (CD31^+^CD45^+^), MDSCs (CD45^+^, Ly6-G(Gr-1)^+^CD11b^+^), and progenitor cells (CD45^+^CD117^+^, CD45^+^Sca-1^+^) 72hrs after myocardial infarction. Data are mean ± SEM. ***p ˂ 0.001.

As previously delineated, WT1 expression was demonstrable in a subset of epicardial cells in normal adult hearts by immunohistochemistry (Figure 1A, D, G). A similar expression pattern was observed in sham-operated animals (in which the ligation around the coronary artery was not closed) (Figure 1B, E, G). However, a significant increase in WT1 expression was detected 72 hours after MI (Figure 1 C, F, G). WT1 co-localized with CD31 (endothelial cells), CD117 and Sca-1 (progenitor cells), CD45 (hematopoietic / myeloid cells), and CD11b and Ly-6G (myeloid-derived suppressor cells) (Figure 2 A-E). In order to quantify WT1 contribution to these different cell populations after acute myocardial infarction, we made use of *Wt1^GFP^* knock in mice where WT1 expression is reflected by cytoplasmic GFP expression 44. Representative double-labelling for GFP and WT1 of *Wt1^GFP^* normal heart and kidney tissues depicts nicely the co-localization in the epicardium of the heart and in kidney podocytes (Figure 2F). Subsequent ImageStream® analyses demonstrated in agreement with our previous findings that the number of WT1 expressing cells in the heart following myocardial infarction is strongly upregulated. There was no difference in cardiac GFP (WT1) expression in normal compared to sham-operated animals (Figure 2G). We next investigated the quantitative contribution of GFP (WT1) positive cells to the different cell populations identified before in immunostainings. WT1 (GFP) marked the majority of endothelial cells (CD31+CD45-) in the heart after myocardial infarction, was expressed in around 50% of the progenitor cell populations and more than half of MDSCs (CD45^+^Ly6-G^+^CD11b^+^) (Figure 2H, Figure S1A-E).

### Conditional knockout of WT1 induces hypertrophy and reduces cardiac function in the acute phase after MI

**Figure 3:**
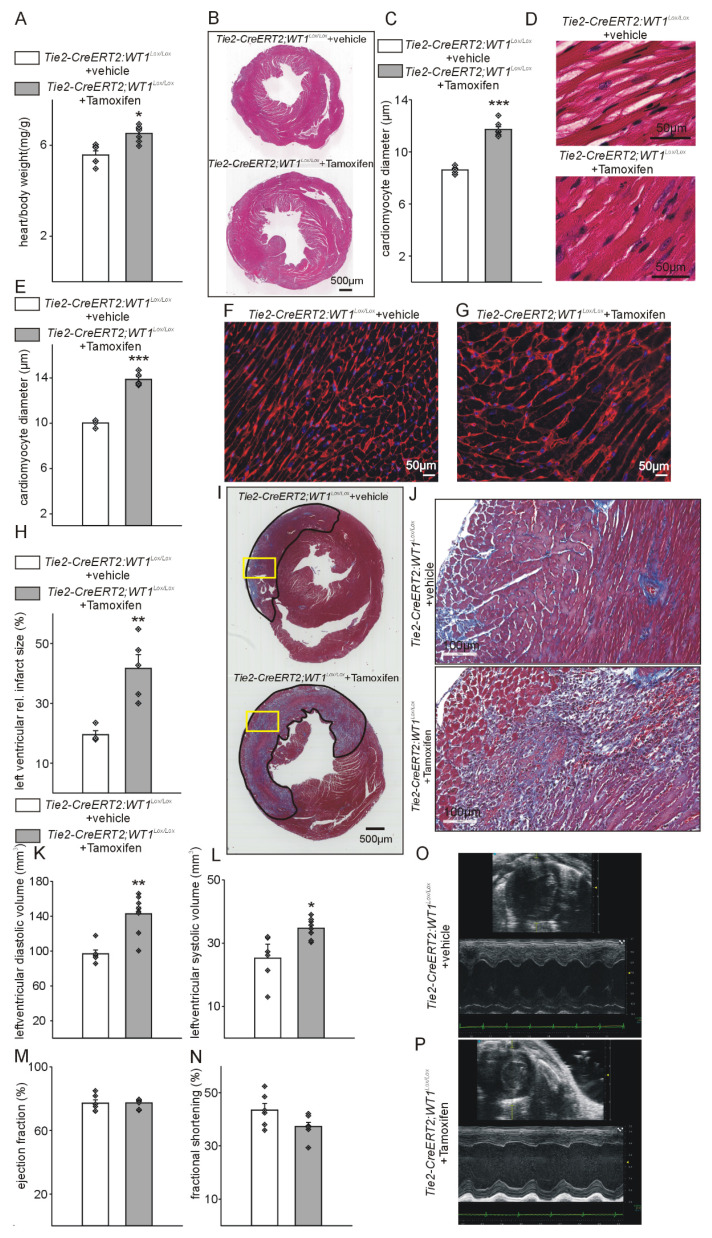
** Tie2-CreERT2-mediated conditional WT1 knockout leads to additional cardiac hypertrophy, enhanced tissue damage, and worsened functional parameters compared to controls shortly after MI.** Respective heart-to-body weight ratios (A), photomicrographs of HE-stained heart sections (B), quantification of cardiomyocyte diameters based on measurements from HE-stained heart sections (C), and high-power photomicrographs of HE-stained heart sections showing individual cardiomyocytes (D) from *Tie2-CreERT2;Wt1^lox/lox^*+vehicle controls (*n =* 6) and *Tie2-CreERT2;Wt1^lox/lox^*+Tamoxifen mice (*n =* 7). (E) Quantification of cardiomyocyte diameters based on measurements from WGA-stained heart sections, and high-power photomicrographs of WGA-stained heart sections showing individual cardiomyocytes (F) from *Tie2-CreERT2;Wt1^lox/lox^*+vehicle controls (*n =* 4) and (G) *Tie2-CreERT2;Wt1^lox/lox^*+Tamoxifen mice (*n =* 5). (H) Quantification of relative left ventricular infarct sizes from *Tie2-CreERT2;Wt1^lox/lox^*+vehicle (*n =* 4), and *Tie2-CreERT2;Wt1^lox/lox^*+Tamoxifen mice (*n =* 5). (I) Representative photomicrographs of Trichrome-Masson-stained heart sections with demarcation of the infarcted area. The yellow rectangles indicate the region of the high-power magnifications. (J) High power photomicrographs of Trichrome-Masson-stained heart tissue sections showing enhanced immune infiltration of *Tie2-CreERT2;Wt1^lox/lox^*+Tamoxifen hearts (lower panel) after MI compared to controls (upper panel). Left-ventricular diastolic volume (K), left-ventricular systolic volume (L), ejection fraction (M), and fractional shortening (N) as echocardiographic parameters of *Tie2-CreERT2;Wt1^lox/lox^*+vehicle) (*n =* 6*)* and* Tie2-CreERT2*+Tamoxifen (*n =* 8*)* animals after MI. Representative echocardiographic images for (O) *Tie2-CreERT2;Wt1^lox/lox^*+vehicle, and (P) *Tie2-CreERT2;Wt1^lox/lox^*+Tamoxifen animals. Data are mean ± SEM. *p ˂ 0.05; **p ˂ 0.01.

To characterize the function of WT1 in endothelial and hematopoietic-derived / myeloid cells after MI, we used WT1 conditional knockout mice (*Wt1^lox/lox^*) crossed with a Tamoxifen-inducible Cre line, which is active in endothelial and hematopoietic-derived / myeloid cells (*Tie2-CreERT2*). Recombination and knockout of WT1 is efficient in endothelial cells and hematopoietic-derived cells of the resulting mouse line (*Tie2-CreERT2;Wt1^lox/lox^*+Tamoxifen) 32. Heart-to body weight ratios (Figure S2A, B) were comparable and echocardiography (Figure S2C-I) did not reveal significant differences in cardiac function in adult healthy animals with knockout of WT1 in in endothelial and hematopoietic-derived cells (*Tie2-CreERT2;Wt1^lox/lox^*+Tamoxifen) compared to the respective control groups. In animals with WT1 knockout in endothelial and hematopoietic-derived cells, already in the acute phase after MI, heart-to body weight ratios were increased and cardiomyocyte diameters determined by measurements of HES and WGA-stained sections higher indicating cardiac hypertrophy (Figure 3A-G). Infarct sizes were bigger and higher immune infiltration of the infarcted areas marked animals with knockout of WT1 in in endothelial and hematopoietic-derived cells (Figure 3I, J). Echocardiographic measurements revealed enlarged cardiac systolic and diastolic dimensions in these animals compared to the respective control group (Figure 3K-P).

**Figure 4:**
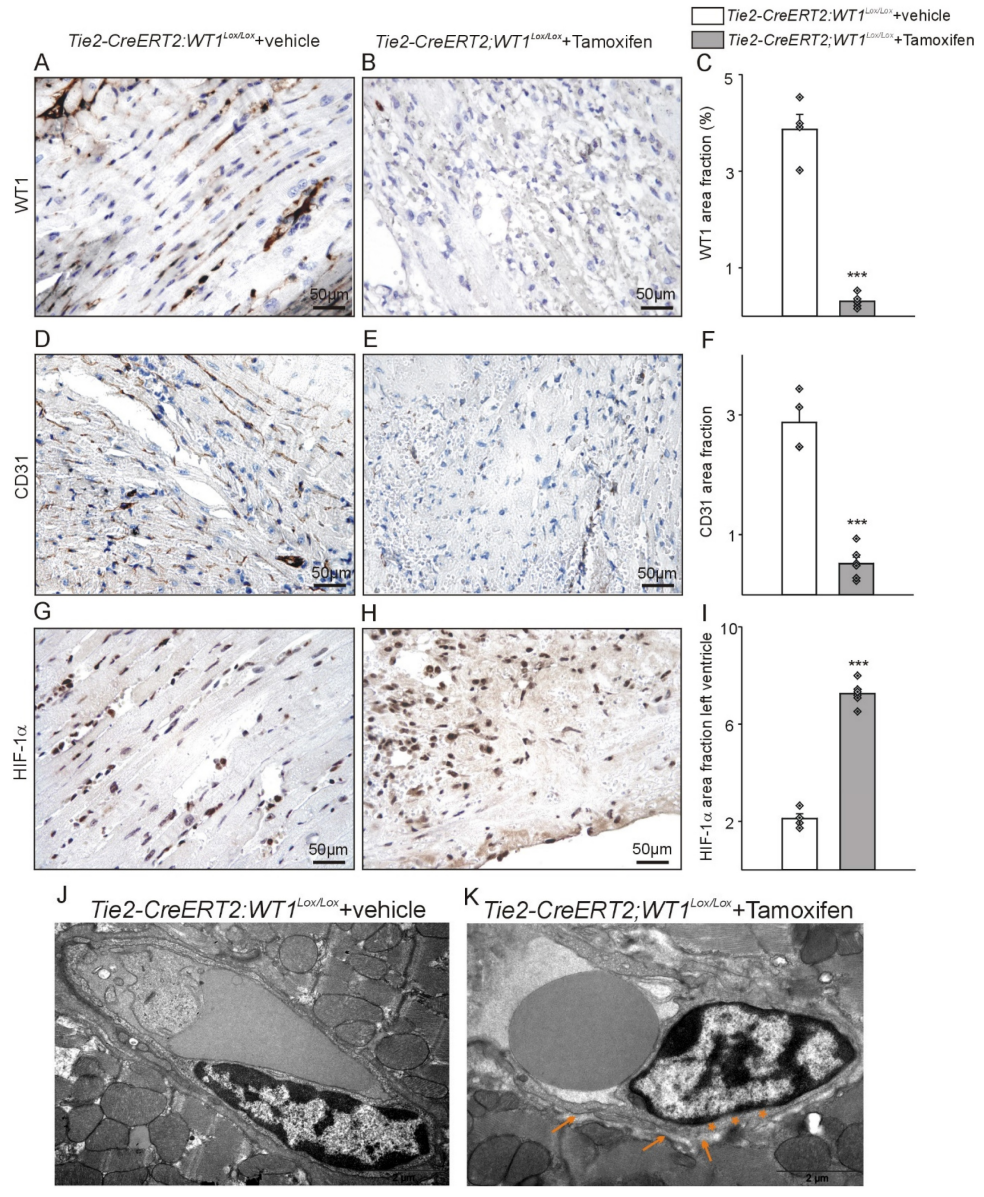
** Tie2-CreERT2-mediated knockout of WT1 in endothelial and hematopoietic-derived cells impairs angiogenesis, increases hypoxia, and damages vessel integrity in the acute phase after MI.** Photomicrographs of WT1 immunostained left ventricles after acute MI of (A) *Tie2-CreERT2;Wt1^lox/lox^*+vehicle and (B) *Tie2-CreERT2;Wt1^lox/lox^*+Tamoxifen animals. (C) Quantification of WT1 signal area fractions of left ventricle sections from *Tie2-CreERT2;Wt1^lox/lox^*+vehicle controls (*n =* 4) and *Tie2-CreERT2*+Tamoxifen (*n =* 6*)* animals after acute MI. Photomicrographs of CD31 immunostained left ventricles after acute MI of (D) *Tie2-CreERT2;Wt1^lox/lox^*+vehicle and (E) *Tie2-CreERT2;Wt1^lox/lox^*+Tamoxifen animals. (F) Quantification of CD31 signal area fractions of left ventricle sections from *Tie2-CreERT2;Wt1^lox/lox^*+vehicle controls (*n =* 4) and *Tie2-CreERT2*+Tamoxifen (*n =* 6*)* animals after acute MI. Photomicrographs of HIF-1α immunostained left ventricles after acute MI of (G) *Tie2-CreERT2;Wt1^lox/lox^*+vehicle and (H) *Tie2-CreERT2;Wt1^lox/lox^*+Tamoxifen animals. (I) Quantification of HIF-1 α signal area fractions of left ventricle sections from *Tie2-CreERT2;Wt1^lox/lox^*+vehicle controls (*n =* 4) and *Tie2-CreERT2*+Tamoxifen (*n =* 6*)* animals after acute MI. Representative high resolution electron microscopy images of left ventricular vessels of (J) *Tie2-CreERT2;Wt1^lox/lox^*+vehicle controls (n = 3) and (K) *Tie2-CreERT2;Wt1^lox/lox^*+Tamoxifen mice (n = 3). Note the starting degradation of the endothelial cell basement membrane (orange asterisks) and the enlargement of the subcellular space (orange arrows) in the *Tie2-CreERT2;Wt1^lox/lox^*+Tamoxifen animal. Data are mean ± SEM. ***p ˂ 0.001.

WT1 immunohistochemistry followed by image quantification confirmed reduced WT1 expression in *Tie2-CreERT2;Wt1^lox/lox^*+Tamoxifen animals. CD31 immunohistochemistry revealed diminished vessel density in *Tie2-CreERT2;Wt1^lox/lox^*+Tamoxifen animals compared to controls. Most likely as a result of impaired angiogenesis, hypoxia as indicated by HIF-1α staining, was more pronounced in the left ventricles of *Tie2-CreERT2;Wt1^lox/lox^*+Tamoxifen mice after MI compared to controls (Figure 4A-I). Electron microscopy revealed a beginning degradation of the endothelial cell basement membrane and enlargement of the subcellular space in *Tie2-CreERT2;Wt1^lox/lox^*+Tamoxifen animals compared to controls (Figure 4J, K).

**Figure 5:**
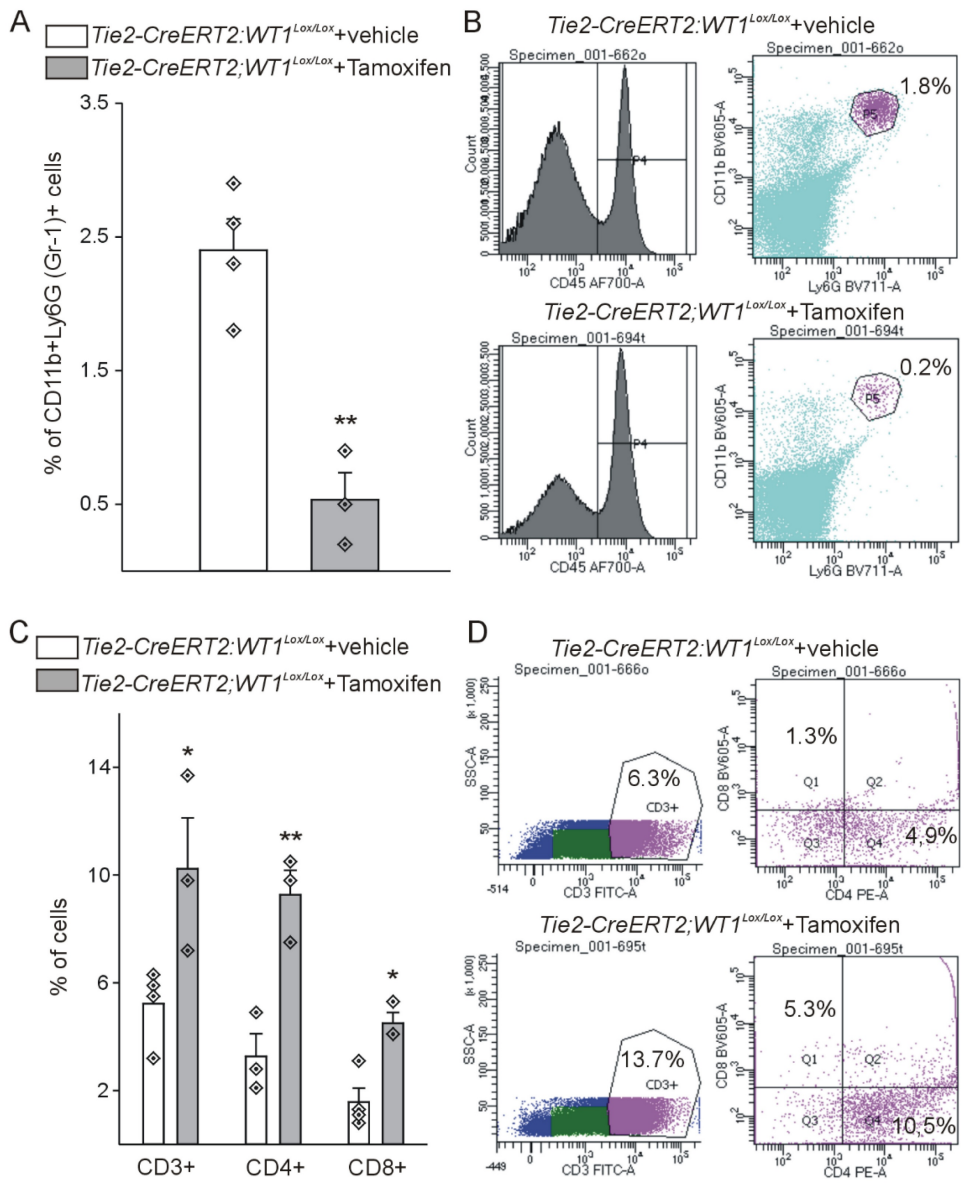
** Cardiac lesions of *Tie2-CreERT2;Wt1^lox/lox^*+Tamoxifen animals in the acute phase after MI are characterized by less MDSC infiltration and higher lymphocyte invasion.** (A) Quantification of flow cytometry analysis of the fraction of CD45^+^CD11b^+^Ly-6G (Gr-1)^+^ MDSCs from hearts of *Tie2-CreERT2;Wt1^lox/lox^*+vehicle controls (*n =* 4) and *Tie2-CreERT2;Wt1^lox/lox^*+Tamoxifen (*n =* 3*)* animals after acute MI. Representative flow cytometry examples (B) of a *Tie2-CreERT2;Wt1^lox/lox^*+vehicle control heart (upper panel) and a *Tie2-CreERT2;Wt1^lox/lox^*+Tamoxifen heart (lower panel) for MDSC quantification. (C) Quantification of flow cytometry analysis of the fraction of CD3^+^, CD3^+^CD4^+^, and CD3^+^CD8^+^ lymphocytes from hearts of *Tie2-CreERT2;Wt1^lox/lox^*+vehicle controls (*n =* 4) and *Tie2-CreERT2;Wt1^lox/lox^*+Tamoxifen (*n =* 3*)* animals after acute MI. Representative flow cytometry examples (D) of a *Tie2-CreERT2;Wt1^lox/lox^*+vehicle control heart (upper panel) and a *Tie2-CreERT2;Wt1^lox/lox^*+Tamoxifen heart (lower panel) for lymphocyte quantification. Data are mean ± SEM. *p ˂ 0.05; **p ˂ 0.01.

Expression of the progenitor cell marker CD117 (c-kit) was lower in *Tie2-CreERT2;Wt1^lox/lox^*+Tamoxifen hearts (Figure S3A-C), which is in agreement with the reported direct regulation of CD117 by WT1 32. CD45 as pan-hematopoietic marker was increased in *Tie2-CreERT2;Wt1^lox/lox^*+Tamoxifen animals with MI (Figure S3D-F). Interestingly, we observed a higher number of CD3-positive T-lymphocytes in these mice (Figure S3G-I). WT1 is expressed in immunosuppressive MDSCs 43,45. We therefore investigated MDSC infiltration using the combination of CD11b / Ly-6G as marker for mouse MDSCs. Both markers were downregulated in the hearts of *Tie2-CreERT2;Wt1^lox/lox^*+Tamoxifen animals with MI and double-stainings revealed a significant reduction of MDSCs (CD11b^+^Ly-6G^+^) (Figure S3J-R). Flowcytometric analysis of *Tie2-CreERT2;Wt1^lox/lox^*+Tamoxifen hearts in the acute phase after infarction revealed on average a nine-fold reduction of MDSC numbers as compared to controls and doubled lymphocyte invasion (including CD3, CD4, and CD8 lymphocytes) (Figure 5).

To determine if the severe phenotype observed after myocardial infarction in *Tie2-CreERT2;Wt1^lox/lox^*+Tamoxifen animals was solely due to diminished vessel formation or to the combination of reduced vascularization with enhanced immune invasion , we examined the effects of conditional deletion of Wt1 restricted to endothelial cells only by using an additional Tamoxifen-inducible endothelial specific Cre line (*VE-cadherin-CreERT2* (*VE-CreERT2*)). Recombination and knockout of WT1 is equally efficient in endothelial cells of both resulting mouse lines (VE*-CreERT2;Wt1^lox/lox^*+Tamoxifen and *Tie2-CreERT2;Wt1^lox/lox^*+Tamoxifen) 43. Echocardiography did not reveal significant differences in cardiac function in adult healthy animals with knockout of WT1 in endothelial cells (VE*-CreERT2;Wt1^lox/lox^*+Tamoxifen), compared to the respective control groups (Figure S4A-G). In the acute phase (48-72 hours) after MI, we did not detect significant differences in heart- to-body weight ratios, histology, cardiomyocyte diameters, and infarct sizes (Figure S5A-I) in VE*-CreERT2;Wt1^lox/lox^*+Tamoxifen animals compared to the respective controls while CD31 immunostaining revealed a significant reduction of left ventricular vessel density in animals with endothelial-specific knockout of WT1 after MI (Figure S5J-L). Despite diminished angiogenesis, cardiac dimensions and function were not significantly different in these animals compared to controls (Figure S5M-R) although we can't exclude that subtle differences, which are not detectable in echocardiography might exist. This model supports previous evidence for a cell-autonomous function of WT1 in endothelial cells for angiogenesis. Nevertheless, enhanced immune invasion combined with reduced angiogenesis after myocardial infarction are more likely to account for the severe phenotype observed in animals with conditional Wt1 knockout in endothelial, hematopoietic-derived and myeloid cells.

### Conditional knockout of WT1 impairs repair after MI

**Figure 6:**
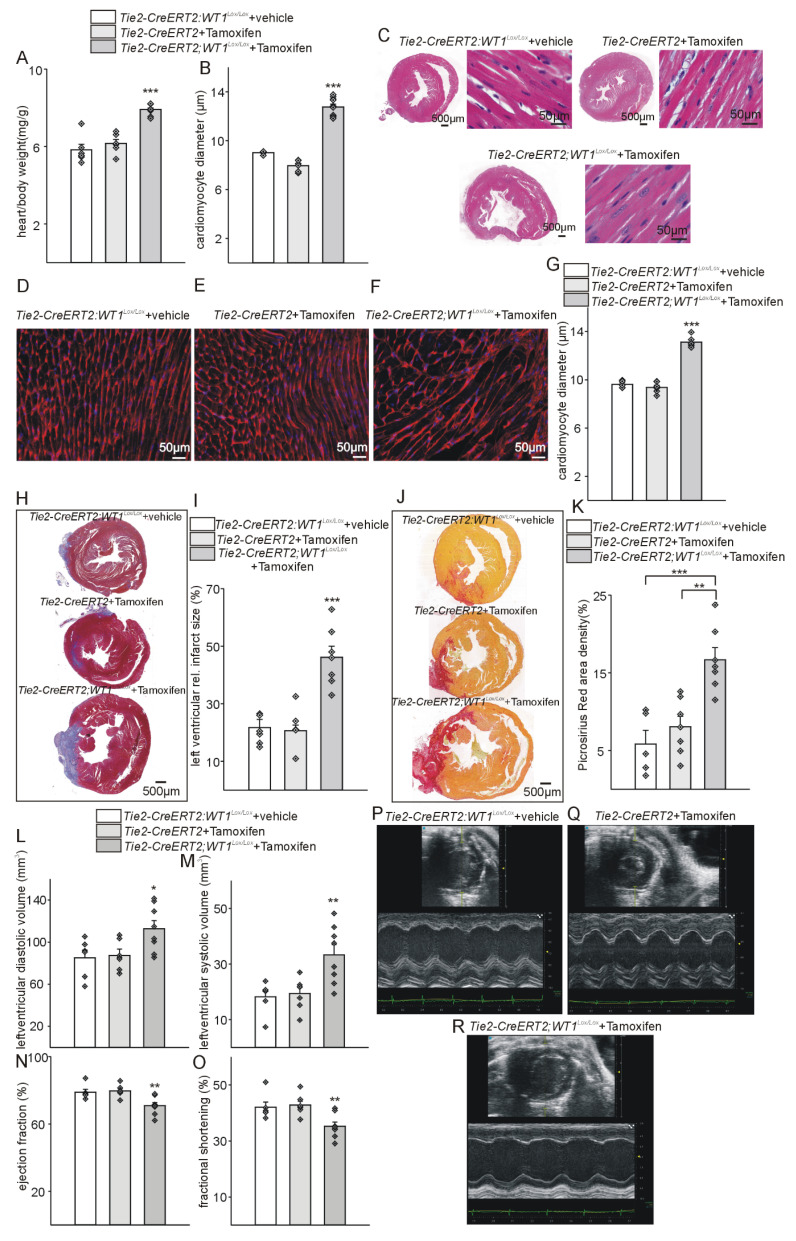
** Tie2-Cre mediated inducible knockout of WT1 leads to cardiac hypertrophy, increased tissue damage, higher cardiac fibrosis, and impaired cardiac function after myocardial infarction.** Heart-to-body weight ratios (A) and quantification of cardiomyocyte diameters based on measurements from HE-stained heart sections (B) from *Tie2-CreERT2;Wt1^lox/lox^*+vehicle (*n =* 6), Tie2-CreERT2+Tamoxifen (*n =* 6) and *Tie2-CreERT2;Wt1^lox/lox^*+Tamoxifen mice (*n =* 10). (C) Photomicrographs of HE-stained heart sections (left panels) and high-power photomicrographs (right panels) of HE-stained heart sections showing individual cardiomyocytes from *Tie2-CreERT2;Wt1^lox/lox^*+vehicle, Tie2-CreERT2+Tamoxifen, and *Tie2-CreERT2;Wt1^lox/lox^*+Tamoxifen mice. High-power photomicrographs of WGA-stained heart sections showing individual cardiomyocytes from (D) *Tie2 -CreERT2;Wt1^lox/lox^*+vehicle (*n =* 5), (E) Tie2-CreERT2+Tamoxifen (*n =* 6), and (F) *Tie2-CreERT2;Wt1^lox/lox^*+Tamoxifen mice (*n =* 6), and quantification of cardiomyocyte diameters based on measurements from the respective WGA-stained heart sections (G). (H) Representative photomicrographs of Trichrome-Masson-stained heart sections with demarcation of the infarcted area (blue staining). (I) Quantification of relative left ventricular infarct sizes from *Tie2-CreERT2;Wt1^lox/lox^*+vehicle (*n =* 6), Tie2-CreERT2+Tamoxifen (*n =* 6), and *Tie2-CreERT2;Wt1^lox/lox^*+Tamoxifen mice (*n =* 7). (J) Photomicrographs of Picrosirius Red stained cross sections and (K) quantification of cardiac fibrosis from *Tie2-CreERT2;Wt1^lox/lox^*+vehicle (*n =* 5), Tie2-CreERT2+Tamoxifen (*n =* 7), and *Tie2-CreERT2;Wt1^lox/lox^*+Tamoxifen mice (*n =* 7). Left-ventricular diastolic volume (L), left-ventricular systolic volume (M), ejection fraction (N), and fractional shortening (O) as echocardiographic parameters of *Tie2-CreERT2;Wt1^lox/lox^*+vehicle (*n =* 6), Tie2-CreERT2+Tamoxifen (*n =* 7), and *Tie2-CreERT2;Wt1^lox/lox^*+Tamoxifen mice (*n =* 8) 3 weeks after MI. Representative echocardiographic images for *Tie2-CreERT2;Wt1^lox/lox^*+vehicle (P), Tie2-CreERT2+Tamoxifen (Q), and *Tie2-CreERT2;Wt1^lox/lox^*+Tamoxifen (R) animals 3 weeks after MI. Data are the mean ± SEM. **p ˂ 0.01; ***p ˂ 0.001.

Given the striking differences in *Tie2-CreERT2;Wt1^lox/lox^*+Tamoxifen mice compared to the respective controls in the acute phase after MI, we further extended the analyses to additional animals of the same genotype in the reparation phase (3 weeks) after myocardial infarction. Heart-to-body weight ratios and cardiomyocyte diameters were significantly higher in *Tie2-CreERT2;Wt1^lox/lox^*+Tamoxifen animals compared to the control groups also in the reparation phase after MI (Figure 6A-G). Trichome Masson and SiriusRed stainings and quantifications revealed increased infarct sizes and fibrosis (Figure 6H-K); and echocardiographic measurements showed higher systolic and diastolic dimensions and reduced ejection fractions and fractional shortening in the animals with WT1 knockout in endothelial and hematopoietic-derived cells compared to controls (Figure 6L-R).

In the reparation phase after MI, WT1 expression was highest in the infarct zone, followed by the border zone, and lowest in the remote zone of the left ventricle in control animals. In all regions of the left ventricle, WT1 expression was significantly lower in *Tie2-CreERT2;Wt1^lox/lox^*+Tamoxifen mice (Figure S6A-F). In contrast, vessel density as indicated by CD31 immunostaining was only significantly reduced in the infarct and border zone in animals with WT1 knockout in endothelial and hematopoietic-derived cells compared to controls while the remote zone was unaffected and the regular architecture between cardiomyocytes and capillaries maintained (Figure S6G-L).

**Figure 7:**
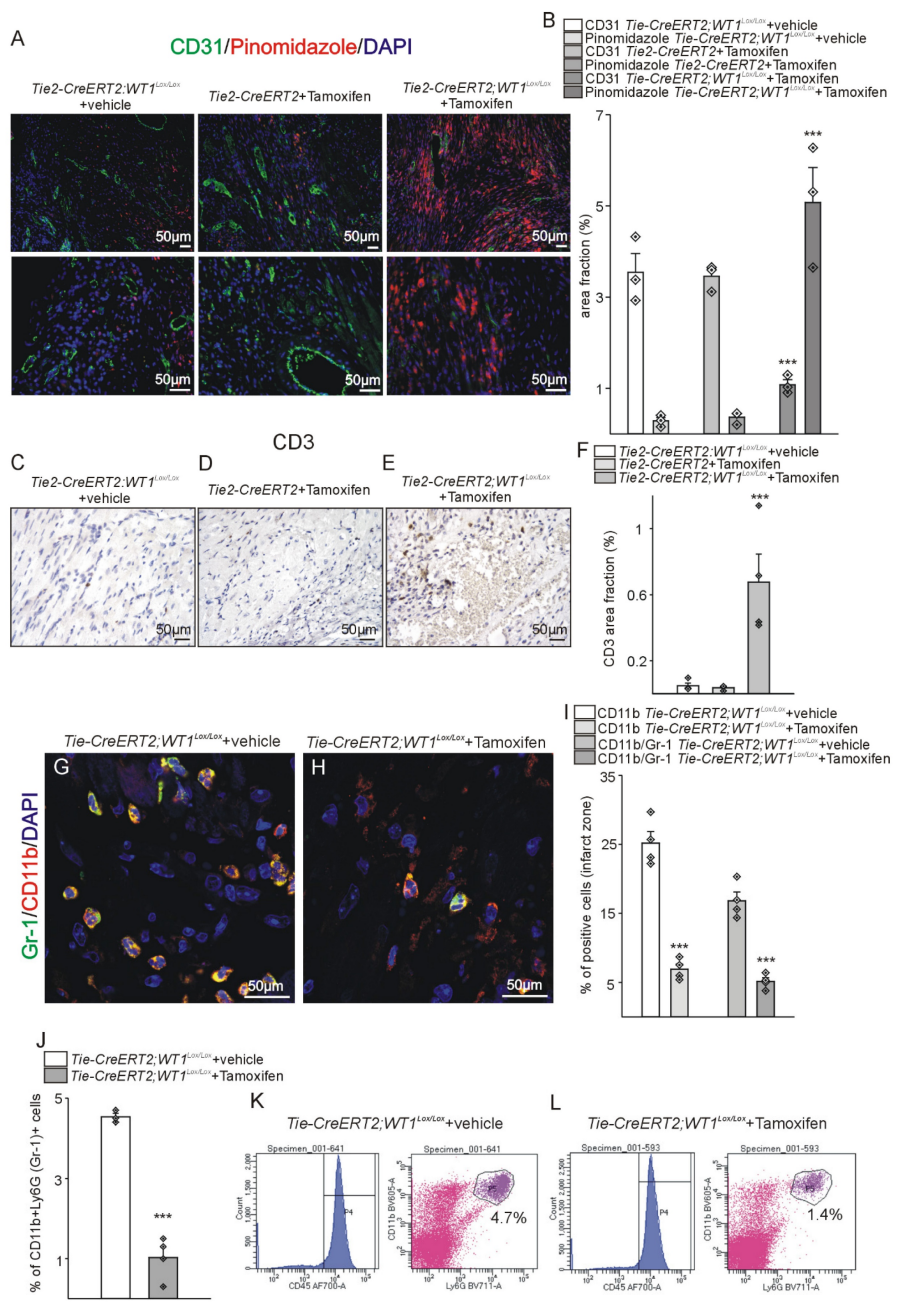
** Expanded hypoxia, increased lymphocyte invasion, and less MDSCs in *Tie2-CreERT2;Wt1^lox/lox^*+Tamoxifen hearts after MI.** (A) Pinomidazole/CD31 double-labelling of *Tie2-CreERT2;Wt1^lox/lox^*+vehicle (left panel), *Tie2-CreERT2*+Tamoxifen (middle panel), and *Tie2-CreERT2;Wt1^lox/lox^*+Tamoxifen (right panel) left cardiac ventricles 3 weeks after MI. Pimonidazole (red) marks hypoxic areas in the heart. CD31 (green) was used to visualize vessels. DAPI (blue) stains nuclei. (B) Quantification of the area fraction for both signals in hearts from *Tie2 -CreERT2;Wt1^lox/lox^*+vehicle controls (*n =* 3), *Tie2-CreERT2*+Tamoxifen (*n =* 3), and *Tie2-CreERT2;Wt1^lox/lox^*+Tamoxifen (*n =* 3) animals 3 weeks after MI. Photomicrographs of CD3 immunostained left ventricles 3 weeks after MI of (C) *Tie2-CreERT2;Wt1^lox/lox^*+vehicle, (D) *Tie2-CreERT2*+Tamoxifen, and (E) *Tie2-CreERT2;Wt1^lox/lox^*+Tamoxifen animals. (F) Quantification of CD3 signal area fractions of left ventricle sections from *Tie2-CreERT2;Wt1^lox/lox^*+vehicle controls (*n =* 4), *Tie2-CreERT2*+Tamoxifen (*n =* 4), and *Tie2-CreERT2;Wt1^lox/lox^*+Tamoxifen (*n =* 4*)* animals. Representative confocal images of CD11b (red) and Ly-6G (Gr-1) (green) double-labelling in the left-ventricular infarct zone from (G) *Tie2-CreERT2;Wt1^lox/lox^*+vehicle and (H) *Tie2-CreERT2;Wt1^lox/lox^*+Tamoxifen animals. DAPI (blue) served as counterstain. (I) Quantification of left-ventricular relative cell numbers positive for CD11b alone or double positive for CD11b and Ly-6G (Gr-1) of *Tie2-CreERT2;Wt1^lox/lox^*+vehicle controls (*n =* 4) and *Tie2-CreERT2;Wt1^lox/lox^*+Tamoxifen (*n =* 4*)* animals 3 weeks after MI. (J) Quantification of flow cytometry analysis of the fraction of CD45+CD11b+Ly-6G (Gr-1)+MDSCs from hearts of *Tie2-CreERT2;Wt1^lox/lox^*+vehicle controls (*n =* 4) and *Tie2-CreERT2;Wt1^lox/lox^*+Tamoxifen (*n =* 4*)* animals 3 weeks after MI. Representative flow cytometry data (K) of a *Tie2-CreERT2;Wt1^lox/lox^*+vehicle control heart and (L) a *Tie2-CreERT2;Wt1^lox/lox^*+Tamoxifen heart for MDSC quantification. Data are mean ± SEM. ***p ˂ 0.001.

In the reparation phase after MI, expression of Hif-1α, which correlates with reduced oxygen supply of the tissue, was highest in the infarct zone followed by the border zone in *Tie2-CreERT2;Wt1^lox/lox^*+Tamoxifen mice and in both regions significantly higher than in the control groups. In the remote zone, no significant differences in Hif-1α expression were detectable between the groups (Figure S7). To further support the finding of increased hypoxia in the left ventricles in *Tie2-CreERT2;Wt1^lox/lox^*+Tamoxifen mice in the reparation phase after MI, additional animals were injected with pimonidazole and double-staining with CD31 performed. In contrast to the control groups, animals with WT1 knockout in endothelial and hematopoietic-derived cells showed reduced angiogenesis (CD31 area fraction) and bigger hypoxic areas (Figure 7A, B). In addition, immunostaining for CD3 and flowcytometric analysis of *Tie2-CreERT2;Wt1^lox/lox^*+Tamoxifen hearts revealed increased lymphocyte invasion as compared to controls (Figure 7C-F, and Figure S8A, B). Histological analysis of Ly-6G and CD11b demonstrated reduced numbers of CD11b and Ly-6G positive cells (Figure S8C-F). Double-labelling for CD11b and Ly-6G and additional FACS analysis confirmed the significant reduction in MDS cell numbers in *Tie2-CreERT2;Wt1^lox/lox^*+Tamoxifen animals compared to controls (Figure 7G-L).

**Figure 8:**
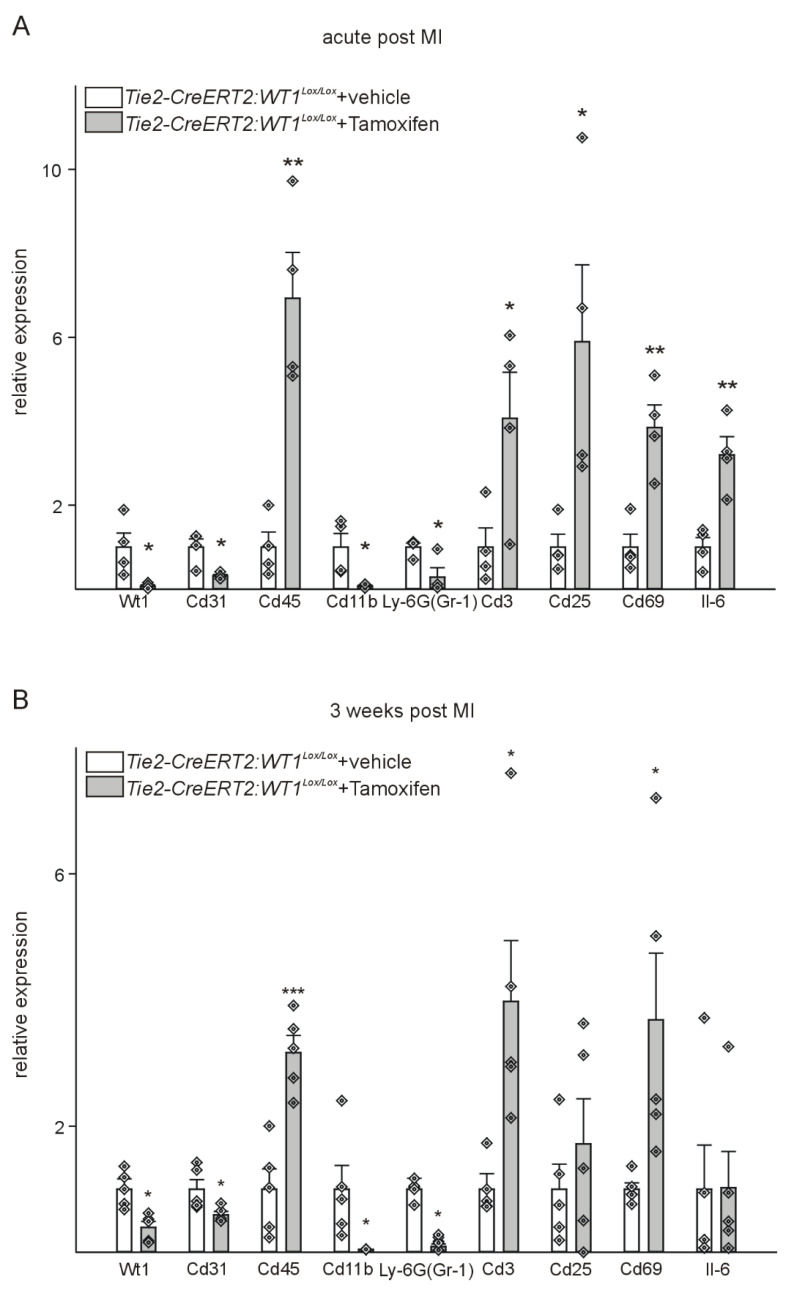
** Tie2-CreERT2-mediated WT1 loss impairs angiogenesis, reduces immunosuppressive mechanisms, and enhances lymphocyte invasion after MI.** Quantitative RT-PCR analyses of markers of angiogenesis and immune function in the acute phase (A) and 3 weeks (B) after MI using *Tie2-CreERT2;Wt1^lox/lox^*+vehicle (*n =* 4) and *Tie2-CreERT2;Wt1^lox/lox^*+Tamoxifen (*n =* 4-5) heart tissues. Expression of each gene was normalized to the respective means of *Gapdh, β-actin*, and *Rplp0* expression. Data are means ± SEM. *p ˂ 0.05; **p ˂ 0.01; ***p ˂ 0.001.

To further support the observed differences, RNA was extracted in the acute and reparative phases after MI, and the expression of WT1, CD31, CD45, CD11b, Ly-6G, CD3, as well as the lymphocyte activation markers Cd25, Cd69 and Il-6 was determined by quantitative PCR. As expected, WT1 expression was significantly lower in *Tie2-CreERT2;Wt1^lox/lox^*+Tamoxifen animals compared to controls at both time points, whereas CD45 as a marker of hematopoietic cells and CD3 as a marker of lymphocytes showed higher expression in both phases. Lymphocyte activation markers Cd25, Cd69, and Il-6 were significantly up-regulated in the acute phase, and Cd69 remained increased at later stages after MI. Animals with knockout of WT1 in endothelial and hematopoietic derived cells exhibited downregulation of CD31, CD11b, and Ly-6G (Figure 8).

**Figure 9:**
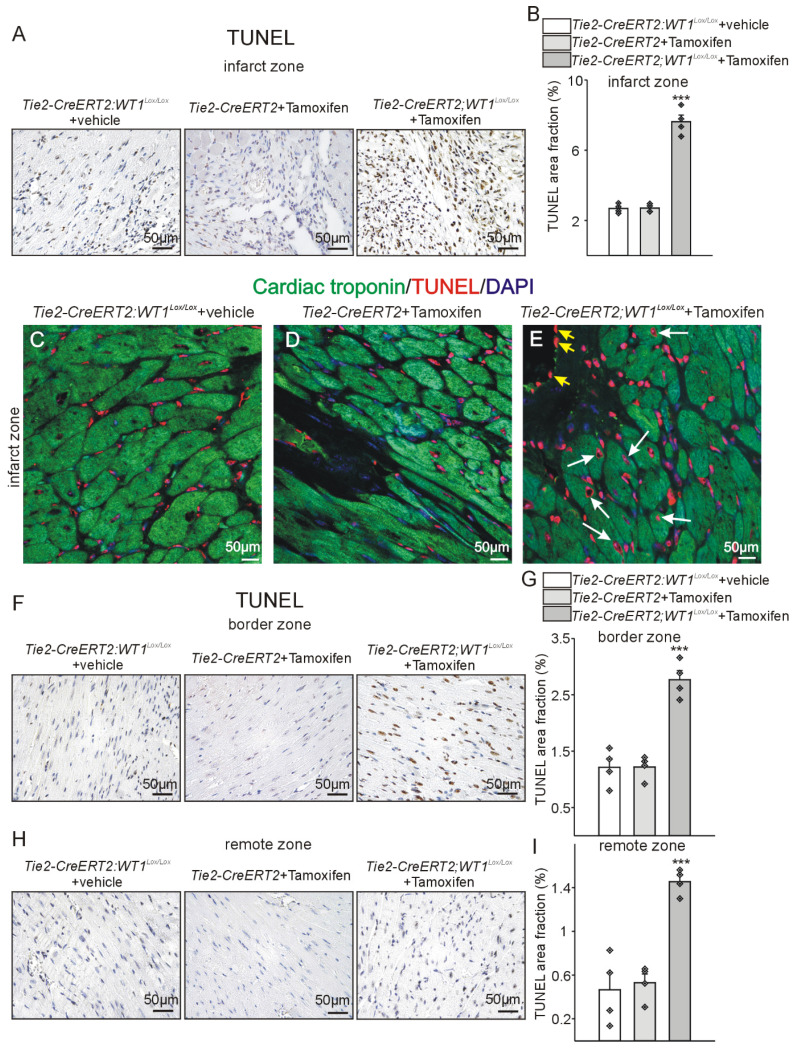
** Higher apoptosis in *Tie2-CreERT2;Wt1^lox/lox^*+Tamoxifen hearts after MI.** Photomicrographs of TUNEL immunostained left ventricles 3 weeks after MI in the infarct (A, C, D, E) border (F), and remote zone (H) of *Tie2-CreERT2;Wt1^lox/lox^*+vehicle, *Tie2-CreERT2*+Tamoxifen, and *Tie2-CreERT2;Wt1^lox/lox^*+Tamoxifen animals. Quantification of TUNEL signal area fractions in the infarct (B), border (G), and remote (I) zone of hearts from *Tie2-CreERT2;Wt1^lox/lox^*+vehicle controls (*n =* 4), *Tie2-CreERT2*+Tamoxifen (*n =* 4), and *Tie2-CreERT2;Wt1^lox/lox^*+Tamoxifen (*n =* 4) animals 3 weeks after MI. Representative confocal images of cardiac troponin (green) and TUNEL (red) double-labelling of the infarct zone from heart sections from *Tie2-CreERT2;Wt1^lox/lox^*+vehicle (C), *Tie2-CreERT2*+Tamoxifen (D), and *Tie2-CreERT2;Wt1^lox/lox^*+Tamoxifen (E) animals. DAPI (blue) served as counterstain of nuclei. White arrows indicate TUNEL positive cardiomyocytes, yellow arrows designate TUNEL positive vascular endothelial cells. Data are mean ± SEM. ***p ˂ 0.001.

As cardiac angiogenesis determines reperfusion after myocardial infarction, which is correlated inversely with apoptotic cell death and apoptosis in the remote zone is associated with reduced cardiac function (reviewed in 46), we reasoned that apoptosis might be affected secondarily to the disturbed angiogenesis and higher immune invasion in animals with conditional knockout of WT1. TUNEL-staining in the reparation phase revealed higher numbers of apoptotic cells in the infarcted, the border, as well as the remote zone in *Tie2-CreERT2;Wt1^lox/lox^*+Tamoxifen animals compared to the control groups (Figure 9). Of note, the number of apoptotic cells was highest in the infarcted area followed by border and remote zone. As revealed by double-labelling for cardiac troponin and TUNEL, many cardiomyocytes were affected by apoptosis (white arrows), but also numerous other stromal cells and endothelial cells of the inner vessel lining (yellow arrowheads) (Figure 9C-E). As a second fundamental cellular mechanism for cardiac repair after MI, we investigated proliferation 6. Immunostaining for PCNA as a marker for proliferating cells revealed lower numbers of PCNA-positive cells in the infarct and border zone but not in the remote zone in *Tie2-CreERT2;Wt1^lox/lox^*+Tamoxifen animals compared to the control groups (Figure S9). Cardiomyocytes in the infarct zone of *Tie2-CreERT2;Wt1^lox/lox^*+Tamoxifen mice were rarely PCNA positive, whereas some proliferating cardiomyocytes could be detected in the respective controls (Figure S9C-E).

### WT1 transcriptionally activates CD11b and Ly-6G

**Figure 10:**
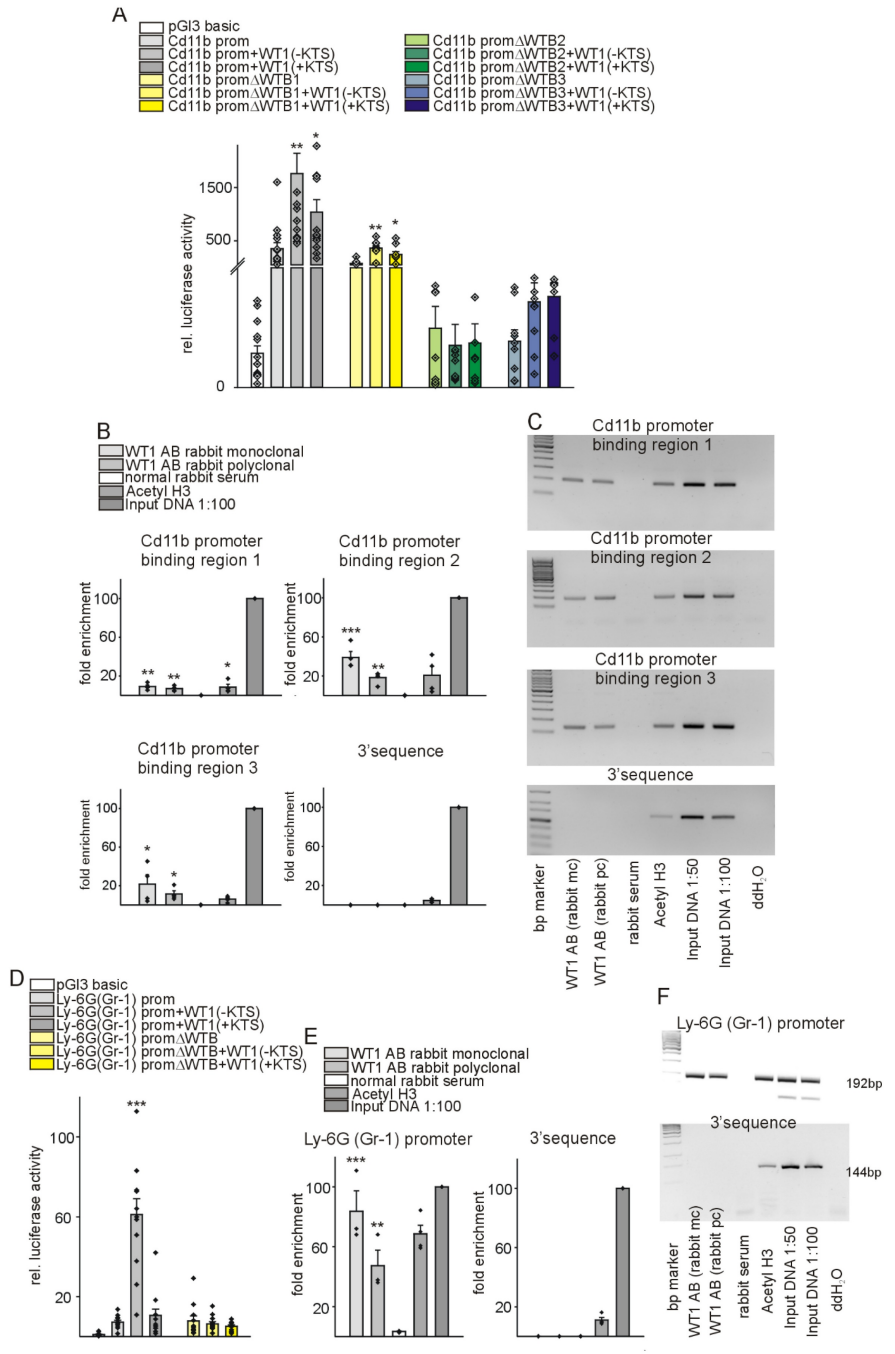
** WT1 binds and transcriptionally activates regulatory sequences of CD11b and Ly-6G, markers of myeloid derived suppressor cells.** (A) Luciferase activity of reporter constructs carrying mouse CD11b promoter in the presence of WT1(-KTS) or WT1(+KTS) expression constructs. Transient transfections were performed using HEK293 cells (*n =* 12 each). The promoterless luciferase expression construct (pGl3basic) served as negative control. ΔWTB indicates reporter constructs with deletion of the predicted WT1-binding sites. (B, C) Chromatin immunoprecipitation (ChIP, *n =* 4) was performed using M15 mouse cell extracts and monoclonal and polyclonal antibodies against WT1 or anti-acetyl-histone H3 antibody. Normal rabbit serum served as negative control. Input DNA was used as a positive control for quantitative PCRs (B) or semiquantitative PCRs ((C), representative agarose gels) for the Cd11b promoter and respective 3′UTR sequences. (D) Luciferase activity of reporter constructs carrying mouse Ly-6G promoter in the presence of WT1(-KTS) or WT1(+KTS) expression constructs. Transient transfections were performed using HEK293 cells (*n =* 12 each). The promoterless luciferase expression construct (pGl3basic) served as negative control. ΔWTB indicates a reporter construct with deletion of the predicted WT1-binding site. For promoter-deletion constructs, the indicated WT1-binding site was removed from the promoter reporter construct. (E, F) Chromatin immunoprecipitation (ChIP, *n =* 4) was performed using M15 mouse cell extracts and monoclonal and polyclonal antibodies against WT1 or anti-acetyl-histone H3 antibody. Normal rabbit serum served as negative control. Input DNA was used as a positive control for quantitative PCRs (e) or semiquantitative PCRs ((f), representative agarose gels) for the Ly-6G promoter and respective 3′UTR sequences. Data are mean ± SEM. *p ˂ 0.05; **p ˂ 0.01; ***p ˂ 0.001.

In a further attempt, we aimed at identifying underlying molecular mechanisms for the observed differences between control animals and the group with knockout of WT1 in endothelial and hematopoietic derived cells. It has been described that WT1 acts downstream of Hif-1α 47 and WT1 transcriptionally activates CD31 and CD117 (c-kit) in vessel formation 43,47. As we observed a significant impact of WT1 deletion on MDSC infiltration and immunosuppressive function after MI, we considered a potential regulation of MDSC-defining molecules by WT1. Thus, we predicted *in silico* potential binding sites of WT1 in the promoter regions of Cd11b and Ly-6g. The Cd11b promoter region contains three putative WT1-binding elements while one was identified in the Ly-6g promoter. Next, we cloned the promoter sequences of Cd11b and Ly-6g in the pGl3basic reporter vector. A schematic illustration of the promoter sequences with the predicted binding sites and position of oligonucleotides used in chromatin immunoprecipitation experiments is provided in Figure S10. Co-transfection of WT1(-KTS) or WT1(+KTS) expression plasmids significantly enhanced the activity of the Cd11b promoter construct in HEK293 cells, which endogenously express low levels of WT1. Deletion of the identified WT1-binding sites reduced the stimulation by co-transfection with WT1 expression plasmids (Figure 10A). Notably, basal activity of Cd11b promoter construct with deletion of WTB2 and WTB3 was lower compared to the wild-type construct suggesting that the basal expression of Cd11b also replies to WT1 in these cultured cells. Chromatin immunoprecipitation with two different WT1 antibodies, an anti-acetyl-Histone H3 antibody as positive control and normal serum as negative control followed by quantitative or semi-quantitative PCRs revealed that WT1 directly binds to all three predicted binding sites in the Cd11b promoter (Figure 10B, C). A comparable approach showed that WT1 also binds and activates the Ly-6g promoter (Figure 10D-F) suggesting that WT1 regulates multiple target genes in cardiac repair.

## Discussion and Conclusion

We demonstrate here that the Wilms' tumor suppressor WT1, expressed in cardiac endothelial and myeloid-derived cells, plays an essential role in post-myocardial infarction outcome in mice. In the adult healthy heart, WT1 is expressed mainly in a subset of epicardial cells, as previously reported 20. Therefore, WT1 has been frequently used to characterize the contribution of epicardial-derived cells to cardiac development and repair 26,30,48,49. The observations that WT1 mRNA and protein could be detected already 24 hours after MI in the deep myocardium 30 and that endothelial-specific knockout of WT1 during embryonic development results in disturbed coronary vessel formation strongly suggest that WT1 has functions in the heart that are independent of the epicardium. In the acute phase following myocardial infarction, we observed a significant re-expression of WT1 in the damaged hearts. This was not observed in sham-operated animals without ligation of the coronary artery, indicating that the effects are not related to the trauma of our minimal invasive surgery. Co-localization with endothelial and progenitor cell markers agrees with previous reports 12,30,43. Notably, WT1 has been described as a direct transcriptional activator of Cd31 (Pecam-1) and Cd117 (c-Kit) 43. In addition, we detected re-expression of WT1 in hematopoietic-derived cells and in myeloid-derived suppressor cells after MI, which is comparable to the situation in tumors in mice and humans 43. Also in zebrafish, a pro-regenerative WT1-positive subtype of immune modulatory cells has been identified 33.

The expression of WT1 has been described previously in cardiomyocytes during embryonic development and reactivation in this cell type following MI 12. Interestingly, WT1 has been shown to maintain cardiomyocyte progenitor cells in a more undifferentiated state in mice and zebrafish 12,17. Also in human failing hearts, WT1 has been detected in a progenitor cell population with proliferative capacity 50. Furthermore, downregulation of WT1 in the heart during the first week after birth 12 has been observed to coincide with withdrawal of cardiomyocytes from the cell cycle, thereby establishing a barrier to cardiac regeneration 51. Consequently, the cardiac response to damage is exacerbated in mice with inducible cardiomyocyte-specific knockout of WT1 31. To investigate the potential function of WT1-expressing endothelial and myeloid-derived cells for cardiac repair after myocardial infarction, we used here conditional WT1-knockout mice crossed either with an inducible endothelial-specific *VE-cadherin-CreERT2* (*VE-CreERT2*) 52 or an inducible Cre line, which is active in endothelial and hematopoietic-derived / myeloid cells (*Tie2-CreERT2*) 53-55. In accordance with the hypothesis that WT1 plays a role in angiogenesis 35-37,42,43, we observed a reduction in cardiac vessel density in both animal models compared to the respective control groups following MI.

Nevertheless, the cardiac phenotype subsequent to myocardial infarction was considerably more severe in animals with endothelial and myeloid-derived cell-specific knockout of WT1 using the Tie2-Cre line, indicating an additional important role for WT1 expressing immune and progenitor cells in cardiac repair following infarction. Besides the reduced vessel density in the infarcted and border region, which was comparable in both models, we detected in the animals with the conditional WT1 knockout in endothelial, hematopoietic-derived / myeloid cells a severe hypoxia in these regions, high Hif-1α expression, and observed a significantly decreased number of MDSCs, increased lymphocyte cell infiltration, cardiac hypertrophy, apoptosis, fibrosis and reduced cardiac function. Increased hypoxia and Hif-1α stabilization might be directly related to the reduced vessel formation in our model 56, which is supported by the observation that the lowest CD31 area fraction and the highest pinomidazole signal, which serves as a marker for hypoxia, were noted in the hearts of Tie2-CreERT2;Wt1^lox/lox^+Tamoxifen animals when compared to the control groups. As Hif-1α activates WT1 47 and WT1 also stimulates VEGF expression 37,38, the cardioprotective and pro-angiogenic functions of Hif-1α after myocardial infarction 56 are reduced in animals with vessel-specific knockout of WT1. In addition, immune status and hypoxia are also closely linked after MI 57 and the observed higher immune cell invasion might further contribute to increased hypoxia in our model. Similar, the cardiac hypertrophy can be in part attributed to enhanced myocardial Hif-1α signaling 58 but might also be caused by the dysbalanced immune phenotype observed in our model 59. Dysregulation of immune cells in the hearts of animals with vessel and myeloid-derived cell-specific knockout of WT1 after MI was characterized by a lower number of CD11b/Ly6G-positive cells (MDSCs) and an increase of CD3-positive lymphocytes in the hearts of Tie2-CreERT2;Wt1^lox/lox^+Tamoxifen animals compared to controls. This finding is consistent with our previous observations that mouse MDSCs express WT1. Furthermore, we showed that wild-type MDSCs suppress T cell proliferation, while WT1-deficient MDSCs loose this function, which corresponds to the higher number of T cells 43. A major focus of research has been to characterize the roles of different monocyte/macrophage populations in cardiac remodeling and repair after MI (reviewed in 60). Relatively limited data exist regarding the role of lymphocytes and MDSCs after myocardial infarction. Nevertheless, several lines of evidence suggest a negative impact of T cells for repair after myocardial infarction: CD4 lymphocytes have deleterious effects in the context of myocardial ischemia/reperfusion injury 61, lymphocyte-deficient mice exhibited reduced infarct sizes 62, and anti-CD3 antibody infusion reduced the scar formation after MI 63, while MDSC infusion or pharmacological stimulation reduced heart failure development by inhibiting a detrimental inflammatory response and cardiomyocyte hypertrophy 64. Therefore, WT1-deficiency might additionally contribute to the increased inflammatory response and hypertrophy by acting in MDSCs. Unfortunately, we were unable to address the question of to what extent WT1-expressing MDSCs exclusively contribute to the repair after MI as to the best of our knowledge, no specific Cre line exist that would allow for the knockout of WT1 solely in MDSCs. Mechanistically, we show that WT1 directly transcriptionally activates CD11b and Ly-6G, which are essential for reducing the inflammatory response 65, maintaining T cell homeostasis 66, and facilitating the recruitment of MDSCs to the site of injury 67. The direct regulation of Ly-6G by WT1 might explain the lower number of MDSCs in our knockout models as Ly-6G contributes to the recruitment of the cells to the sites of injury 67. Whether this is a major underlying mechanism in our system remains to be determined. A limitation of this mechanistic part of our study is the use of a heterologous cell system as to the best of our knowledge, no murine hematopoietic-derived or specific MDSC cell line with high WT1 expression exists. Further research is needed to clarify the exact functions of CD11b and Ly-6G in repair after MI.

In addition to cardiomyocyte hypertrophy, fibrosis represents another critical issue following myocardial infarction. Ischemia-induced cell death stimulates fibroblast proliferation and myofibroblast transformation. This initial reparative fibrosis is essential for preventing rupture of the ventricular wall. However, an excessive fibrotic response and reactive fibrosis in adult mammals will be detrimental by increasing tissue stiffness and further reducing cardiac function 68. It has been shown that cardiac fibroblast activation and myofibroblast transformation are exaggerated by tissue hypoxia following MI 69. Thus, it can be reasonably postulated that the augmented cardiac fibrosis observed in Tie2-CreERT2;Wt1^lox/lox^+Tamoxifen animals following MI is, at least in part, attributable to the prolonged tissue hypoxia in the infarcted and border zone that we have documented. Lymphocyte ablation in juvenile heart injury has been shown to reactivate cardiac regeneration through reduction of fibrosis and enhancement of cardiomyocyte proliferation, again linking enhanced immune invasion to a worsened outcome of ischemic heart disease 70. The enhanced lymphocytic infiltration seen in our model therefore could further add to the significant fibrosis detected.

Both, apoptosis and proliferation after MI, are impacted by the degree of ischemia and hypoxia and immune cell invasion. Hypoxia has been shown to induce HIF-1α, to inhibit proliferation, and to induce cell apoptosis in cardiomyocytes *in vitro* 71. *In vivo*, ablation of lymphocytes decreased apoptosis within the infarcted heart and limited myocardial injury 72. The contribution of apoptotic cell death after MI might determine the final infarct size. Extended apoptosis in the remote zone has been associated with the development of cardiac failure (reviewed in 46). Consequently, the augmented infarct size and diminished cardiac function observed in Tie2-CreERT2;Wt1^lox/lox^+Tamoxifen animals relative to control animals is attributable to the enhanced cardiac tissue hypoxia and lymphocytic invasion and the related increase in the number of apoptotic cells, which we have identified in our model. Furthermore, the diminished proliferation in the infarcted and border zones may contribute to the adverse cardiac remodeling subsequent to myocardial infarction. The collective response of the hearts to MI following the deletion of WT1 in vascular and hematopoietic-derived cells is intricate, and it is hypothesized that this is due to a combination of direct and indirect effects. A comprehensive review of the literature has recently been conducted, offering a thorough examination of the multifaceted roles of immune cells, fibroblasts, cardiomyocytes, and vascular cells in angiogenesis, immune modulation, fibrosis, proliferation, and cardiac repair 60,73-75.

The evidence presented here collectively indicates that the Wilms' tumor suppressor WT1 is essential for multiple stages of cardiac repair, including regulation of post-myocardial infarction neoangiogenesis, immune response, proliferation, and apoptosis. In addition, recent studies have indicated that WT1 in cardiomyocytes plays a role in maintaining the cells in a less differentiated state and is involved in the cardiac response to damage 12,17,31. These findings suggest that WT1 may be a promising target to direct cardiac repair in ischemic heart disease in mammals.

## Methods

### Animals

All animals were used in accordance with local Ethical Committee regulations and approved by the ministry for research (APAFIS #1696-201708281314570, APAFIS #47500-2024031122349766). *Wt1^GFP^* mice 44,76 were used for ImageStream® analyses after myocardial infarctions. Wt1^lox/lox^ and Tie2-CreERT2 animals were crossed to generate Tie2-CreERT2;Wt1^lox/lox^ mice 43,53. VE-cadherin-CreERT2 (VE-CreERT2) mice 52 were crossed with Wt1^lox/lox^ animals to generate the VE-CreERT2;Wt1lox/lox strain 43. Both Cre lines were backcrossed four times with C57BL6. Age- and sex-matched Tie2-CreERT2;Wt1^lox/lox^ or VE-CreERT2;Wt1^lox/lox^ animals were injected for one week intraperitoneally either with sunflower oil (vehicle) or Tamoxifen dissolved in sunflower oil in a dose of 33 mg/kg per day. Tie2-CreERT2 and VE-CreERT2 animals injected with Tamoxifen served as additional controls. Anaesthetized mice were examined by echocardiography 77,78 using the Vevo3100 system with a 55 MHz transducer (Visualsonics, Toronto, Canada). Myocardial infarctions were induced by ligation of the left coronary artery (LAD) as described 78-80. Briefly, anaesthetized mice were endotracheally intubated with a 22G canula, the skin was incised on the left thorax side, the pectoralis muscles were mobilized, a thoracotomy between the third and fourth rib was performed, and the LAD was permanently closed with a 7-0 suture 1mm distal to the left auricle. This resulted in large myocardial infarctions. The thoracotomy and the skin wound were closed with 6-0 sutures and the mice remained intubated until spontaneous respiration was re-established. Lethality of the procedure was approximately 50% due to bleeding, pneumothorax, or arrhythmia mostly immediately after surgery independent of the genotype of the mice.

### Genotyping

The genotype of animals was identified by PCR using the following oligonucleotides and PCR conditions: Cre-F 5′-CGCAGAACCTGAAGATGTTCGCGA-3′; Cre-B 5′-GGATCATCAGCTACACCAGAGACG-3′ (95 °C 3 min, [94 °C 20 s, 60 °C 45 s, 72 °C 1 min] × 27, 72 °C 7 min), Wt1lox-F 5′-TGGGTTCCAACCGTACCAAAGA-3′; Wt1lox-B 5′-GGGCTTATCTCCTCCCATGT-3′ (95 °C 3 min, [93 °C 45 s, 56 °C 45 s, 72 °C 45 s] × 35, 72 °C 7 min).

### RT-PCR and Quantitative RT-PCR

Total RNA was isolated from hearts using the Trizol reagent (ThermoScientific). The RNA pellet was dissolved in diethyl pyrocarbonate-treated H_2_O. First-strand complementary DNA synthesis was performed with 1 μg of total RNA using the RT-Maxima kit (ThermoScientific). One μl of the reaction product was taken for real-time RT-PCR amplification (StepOnePlus, Applied Biosystems) using a commercial SYBR Green kit (ThermoScientific)). Primer sequences are available on request. Expression of each gene was normalized to the respective Gapdh, Actin, and Rplp0 expression.

### Tissue samples, histology, and immunohistology

Histology and measurement of cardiomyocyte diameters were performed according to established protocols 81. Samples from at least five different animals per group (Tie2-CreERT2;WT1^Lox/Lox^ + vehicle, Tie2-CreERT2 + Tamoxifen, Tie2-CreERT2;WT1^Lox/Lox^ + Tamoxifen, VE-CreERT2;WT1^Lox/Lox^ + vehicle, VE-CreERT2 + Tamoxifen, and VE-CreERT2; WT1^Lox/Lox^ + Tamoxifen) were analyzed. Investigators were blinded for the genotype of the mice. Three μm paraffin sections were used for histological and immunohistological procedures.

Haematoxylin-Eosin staining was routinely performed on all tissue samples; additionally, sections were stained with Trichrome Masson and Picrosirius red. Cardiomyocyte diameters were determined on hematoxylin-eosin- or wheat germ agglutinin (WGA)-stained paraffin sections 77. Cardiomyocyte diameters were measured at the level of the nucleus. At least 90 cells per section and three sections per heart were measured. Area fractions for all immunohistological stainings were determined using the ImageJ software. For this purpose, color deconvolution of hematoxylin / DAB-stained slides was performed, the brown (DAB) signal channel selected, the threshold for all samples of a given staining adjusted to the same value, and the area fraction measured 42,43,77,82,83. For quantification of CD31 /Pinomidazole double-staining, single fluorescent channels were selected, and the same quantification strategy applied. Area fractions were analyzed on at least five different sections of hearts per mouse. The number of animals in each group for the different conditions is indicated in the Figure legends. The infarct size was determined on Trichrome Masson-stained sections. For this purpose, 10 different sections below the ligation with an inter-section distance of 200 μm were stained, and the slides scanned. The area of the left ventricle free wall and septum and of the infarct area were determined using the freehand and region of interest measure tools in ImageJ and afterwards the infarct area expressed as percentage of the total ventricle free wall + septum area.

For immunohistology, following heat-mediated antigen retrieval and quenching of endogenous peroxidase activity, antigens were detected using the EnVision Peroxidase/DAB Detection System from Dako (Trappes, France) after antibody application. All samples from the different groups for a given staining and time point were processed at the same time under identical conditions. The color reaction was stopped for all slides in one staining procedure at the same time by washing in water. Sections were counterstained with haematoxylin (Sigma, Saint-Quentin-Fallavier, France). For immunofluorescence double-labelling, sections were incubated with the respective antibodies after preincubation with 10% normal donkey serum in PBS with 1% bovine serum albumin and 0.1% Triton X-100. In case of primary antibodies from mouse, sections were incubated before with mouse blocking reagent (Vector Laboratories). Antigens were visualized using DyLight 488- and DyLight 594-coupled secondary antibodies derived from donkey in a 1:150 dilution (Jackson ImmunoResearch, Newmarket, Suffolk, UK). In case of triple immunostaining, a biotinylated secondary antibody was visualized using AMCA conjugated Avidin D (Vector Laboratories). Omission of the first antibody served as a negative control. In addition, some slides were incubated with immunoglobulin G isotype controls (1:100, rabbit monoclonal, clone SP137 or 1:100, mouse monoclonal, clone MOPC-21, Abcam). The following antibodies were used: WT1 (1:100, mouse monoclonal, clone 6FH2, DAKO); GFP (1:300, rabbit polyclonal, ab290, Abcam), PCNA (1:100, mouse monoclonal, PC10, sc-56, Santa Cruz Biotechnology, Heidelberg, Germany) or (1:100, rabbit monoclonal, ab92552, Abcam); PECAM-1 (CD31) (1:50, rabbit polyclonal, ab28364, Abcam), PECAM-1 (1:20, rat monoclonal, clone SZ31, Dianova, Hamburg, Germany), cardiac troponin T (1:200, mouse monoclonal, clone 1C11, ab8295, Abcam), Pimonidazole (1:200, rabbit polyclonal, HPI Inc.), Hif1α (1:100, rabbit polyclonal, kind gift of J. Pouysségur), CD45 (1:50, rat monoclonal, BD Biosciences, Le Pont de Claix, France), CD117 (1:200, goat polyclonal, AF1356, R&D Systems, Lille, France), CD3 (1:100, rabbit monoclonal, clone SP7, ab16669, Abcam), GR-1 (1:150, rat monoclonal, clone RB 6-805, Biolegend, San Diego, CA, U.S.A), Sca1 (1:50, rat monoclonal, clone D7, Abcam), and CD11b (1:50, rat monoclonal, clone M1/70.15, Biorad, Hercules, CA, U.S.A.) or (1:2000, rabbit monoclonal, ab133357, Abcam). Slides were viewed under an epifluorescence microscope (DMLB, Leica, Germany) connected to a digital camera (Spot RT Slider, Diagnostic Instruments, Scotland).

### TUNEL labelling of apoptotic cells

Apoptotic cells were detected by TUNEL staining of paraffin sections using the *In Situ* Cell Death Detection Kit (Roche Molecular Biochemicals, Meylan, France) as described previously 43.

### Electron microscopy

Mouse hearts were fixed immediately after dissection in 2.5% glutaraldehyde in 0.1 M cacodylate buffer. They were rinsed with buffer and then post-fixed in osmium tetroxide (1%) for 1 h. After rinsing with water, they were dehydrated with acetone and embedded in Epon resin. Eighty-nanometer sections were contrasted with uranyl acetate and lead citrate and then observed in an electron microscope (Philips CM12) operating at 100 kV.

### ImageStream® and Flowcytometry analysis

Hearts were minced and single-cell suspensions were obtained after enzymatic digestion (1 mg ml^-1^ collagenase A and 100 IU ml^-1^ type I DNase (Roche Diagnostics) for 1 h at 37 °C under agitation in serum-free DMEM medium. The single-cell suspension was filtered through a 70-μm cell strainer (BD Biosciences), incubated for three minutes with ammonium-chloride-potassium (ACK) lysing buffer and cells were washed twice in 2% FCS and 0.5 mM EDTA in PBS. After 15 min of saturation in Fc Block (2.4G2) from BD Biosciences, single-cell suspensions were surface stained for 30 min on ice in the dark with the following conjugated antibodies: anti-CD45 (clone 30-F11), anti-CD11b (clone M1/70), anti Ly-6G/Ly-6C (Gr-1) (clone RB6-8C5), anti CD31 (clone 390), anti Ly-6A/E (Sca-1) (clone D7), anti CD117 (clone 2B8), anti CD4 (clone GK1.5), anti CD3 (clone145-2C11), and anti CD8a (clone 53-6.7) from BD Biosciences or BioLegend. Staining was assessed with an ImageStream® or Fortessa flow cytometer, and data were analyzed using either the IDEAS^®^ image analysis software or FacsDiva 6 software.

### Cloning and transient transfection experiments

Promoter sequences of Gr-1 and Cd11b were inspected for potential Wt1-binding sites using the Promo3 software 84. A 1 kb Gr-1 promoter fragment and a 1.6 kb Cd11b promoter part were amplified by PCR from mouse genomic DNA. The promoter constructs were subcloned in the pGl3basic luciferase expression vector (Promega). Each promoter construct was co-transfected with Wt1(-KTS) or Wt1(+KTS) expression constructs in HEK293 cells using Fugene 6 reagent (Promega) (n = 12 each). HEK293 cells were chosen as they express low levels of WT1, are easy to transfect, and in several instances showed the ability to recapitulate *in vivo* regulation 15,35,43. Putative Wt1 binding sites (WTB) were deleted from the promoter constructs using the QuikChange II site directed mutagenesis kit (Stratagene, Agilent Technologies, Massy, France) with the following oligonucleotides: Gr-1, 5'-GTTCACCTTTGGTTTTCAGTGTATCTGGGCAGTAATGTC-3', Cd11bDWTB1, 5'-GGCATGGTTCCACTTGCCCCACATTGTAATAACTTTGAAAC-3', Cd11bDWTB2, 5'- ACTTATTTATTTGGTGCAATGGGTTTCAACTGTGTAAGTATG-3', Cd11bDWTB3, 5'-TCTTTCCTCTTTGGGTATCATGTACCATGGCGTGG-3', each antisense: reverse complement. Deletion constructs were again co-transfected with Wt1(-KTS) or Wt1(+KTS) expression constructs (n = 12 each).

### Chromatin immunoprecipitation assay

Chromatin immunoprecipitation (ChIP) assay was performed on M15 cells using manufacturer's instructions (Millipore) as described 37,43. M15 cells were used in this instance as they are of murine origin and express high levels of endogenous WT1. Normal rabbit serum served as a negative control and dilutions of the input sample as positive control. The following primers were used: Gr-1 promoter, 5'-GCTGAGAGAGCATTACTGAGCA-3' (forward), 5'-CCTAGACCAGACCCCTCCTC-3' (reverse), Gr-1 3'-sequence, 5'-GCAGGGTTCCTATGTGTGCT-3' (forward), 5'-GGCTGAAAGGCCAGAGATGT-3' (reverse), Cd11b promoter WTB1, 5'-TCTGTCTGGTGGCATGGTTC-3' (forward), 5'-GCCTGGCTTCCTTCTCTTCA-3' (reverse), Cd11b promoter WTB2, 5'-CACAGTTGAAACCCACAAGCAT-3' (forward), 5'-TGTGTACATTTCCCTTGGCTGA-3' (reverse), Cd11b promoter WTB3, 5'-CACTATGGCTAGACGCCGAT-3' (forward), 5'-CCCCAGTTTCCATACGGTTGA-3' (reverse), Cd11b 3'-sequence, 5'-AGGAAGGGAGGGGCTTACTT-3' (forward), 5'-CCAATCACCTTAGGGCGACA-3' (reverse). PCR products were electrophoresed on 4% agarose gels. Alternatively, samples were used in quantitative PCRs (n = 4 each).

### Statistical analysis

Data are expressed as means ± S.E.M. Individual values are depicted as dot plots. Analysis of variance (ANOVA) with Bonferroni test as post hoc test was performed for more than two groups. Mann-Whitney tests were performed to compare two groups. The Kolmogrov Smirnov test was used to evaluate whether the data are sampled from populations that follow Gaussian distribution. A p value of <0.05 was considered statistically significant. (GraphPad Instat). All statistical information is provided in the Figure Legends.

### Data availability

Any additional information required to reanalyze the data reported in this work paper is available from the lead contact upon request.

## Supplementary Material

Supplementary figures.

## Abbreviations

CD: cluster of differentiation
ChIP: Chromatin Immunoprecipitation
Hif-1α: hypoxia inducible factor 1 subunit alpha
Ly6G (Gr-1): lymphocyte antigen 6 complex, locus G
MDSCs: myeloid-derived suppressor cells
MI: myocardial infarction
PCNA: proliferating cell nuclear antigen
TUNEL: Terminal Deoxynucleotidyl Transferase-Mediated dUTP Nick End Labeling
VE-CreERT2 mice: VE-cadherin-CreERT2 mice
WT1: Wilms' tumor suppressor 1
