# Supplementary material for: Enhanced neoangiogenesis and balance of the immune response mediated by the Wilms' tumor suppressor WT1 favor repair after myocardial infarction
Source: Theranostics. 2025 Jun 9;15(14):6593–614. doi: 10.7150/thno.104329 (PMC12203559; doi:10.7150/thno.104329)
Supplement: Supplementary file 1 — Supplementary figures. [file thnov15p6593s1.pdf]

Supplementary Material

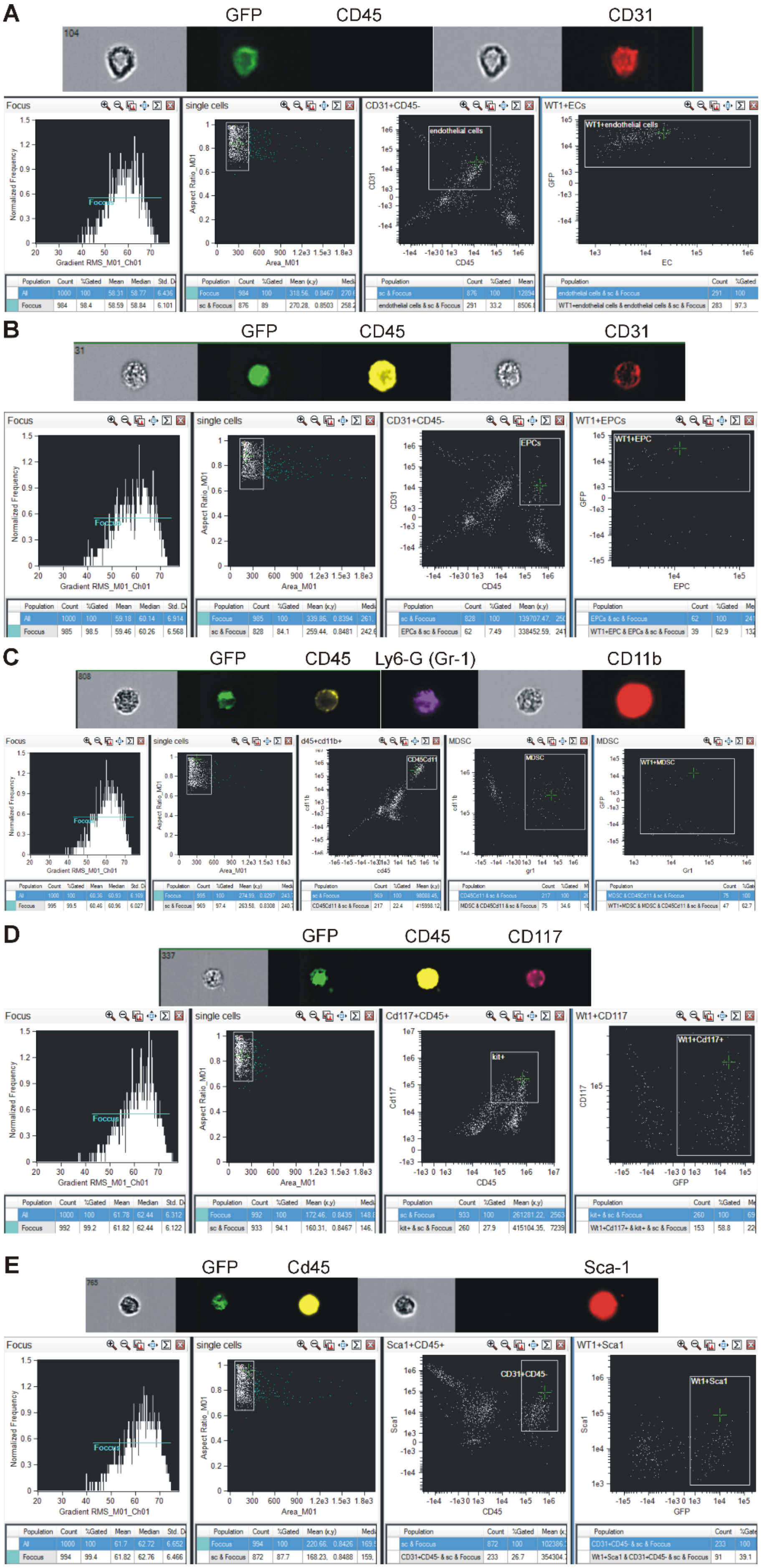
A
GFP
CD45
CD31
Focus
single cells
CD31+CD45-
WT1+ECs
endothelial cells
WT1+endothelial cells
Gradient RMS_M01_Ch01
Area_M01
CD45
EC
B
GFP
CD45
CD31
Focus
single cells
CD31+CD45-
WT1+EPCs
EPCs
WT1+EPC
Gradient RMS_M01_Ch01
Area_M01
CD45
EPC
C
GFP
CD45
Ly6-G (Gr-1)
CD11b
Focus
single cells
d45+cd11b+
MDSC
D
GFP
CD45
CD117
Focus
single cells
Cd117+CD45+
Wt1+CD117
kit+
Wt1+Cd117+
Gradient RMS_M01_Ch01
Area_M01
CD45
GFP
E
GFP
Cd45
Sca-1
Focus
single cells
Sca1+CD45+
WT1+Sca1
CD31+CD45-
Wt1+Sca1
Gradient RMS_M01_Ch01
Area_M01
CD45
GFP

Figure S1: Representative images of ImageStream® multi-labelling cell analysis after myocardial infarction. (A) for $GFP^{+}CD45^{-}CD31^{+}$ cells, (B) $GFP^{+}CD45^{+}CD31^{+}$ cells, (C) $GFP^{+}CD45^{+}$Ly6-G (Gr-1)$^{+}CD11b^{+}$ cells, (D) $GFP^{+}CD45^{+}CD117^{+}$ cells, and (E) $GFP^{+}CD45^{+}$Sca-1^{+} cells.

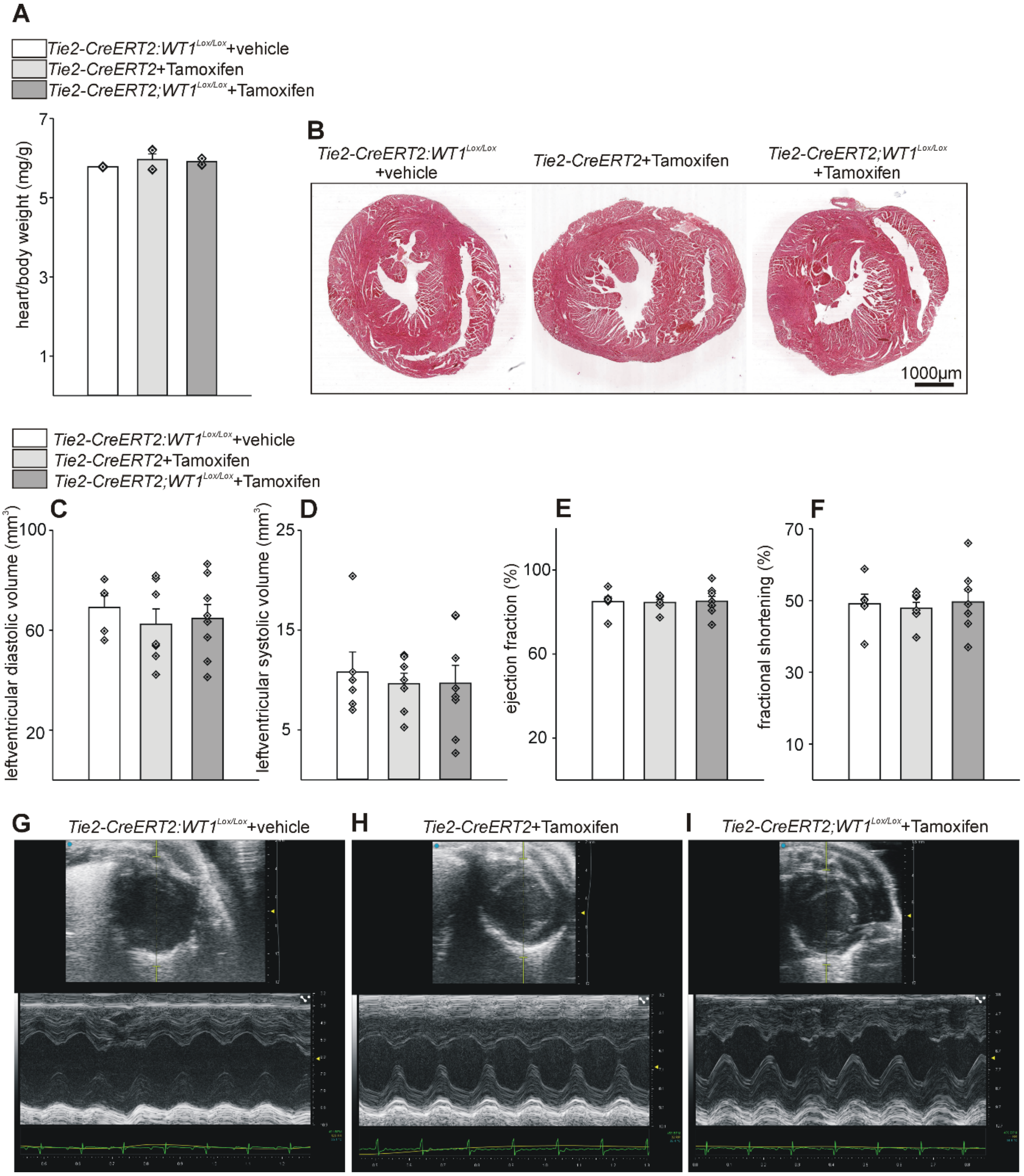

Figure S2: Conditional inducible knockout of WT1 using the Tie2-CreERT2 does not alter heart weights, sizes, or functional parameters. Respective heart-to-body weight ratios (A), and photomicrographs of HE-stained heart sections (B) of *Tie2-CreERT2;Wt1$^{lox/lox}$*+Tamoxifen, *n* = 4; *Tie2-CreERT2;Wt1$^{lox/lox}$*+vehicle, *n* = 4; *Tie2-CreERT2*+Tamoxifen, *n* = 4 animals without MI. Left-ventricular diastolic volume (C), left-ventricular systolic volume (D), ejection fraction (E), and fractional shortening (F) as echocardiographic parameters of *Tie2-CreERT2;Wt1$^{lox/lox}$*+vehicle (*n* = 6*), Tie2-CreERT2*+Tamoxifen (*n* = 7*),* and *Tie2-CreERT2;Wt1$^{lox/lox}$*+Tamoxifen *(n* = 8) animals without MI.

Representative echocardiographic images for (G) *Tie2-CreERT2;Wt1*$^{lox/lox}$+vehicle, (H) *Tie2-CreERT2*+Tamoxifen, and (I) *Tie2-CreERT2;Wt1*$^{lox/lox}$+Tamoxifen animals. Data are mean ± SEM.

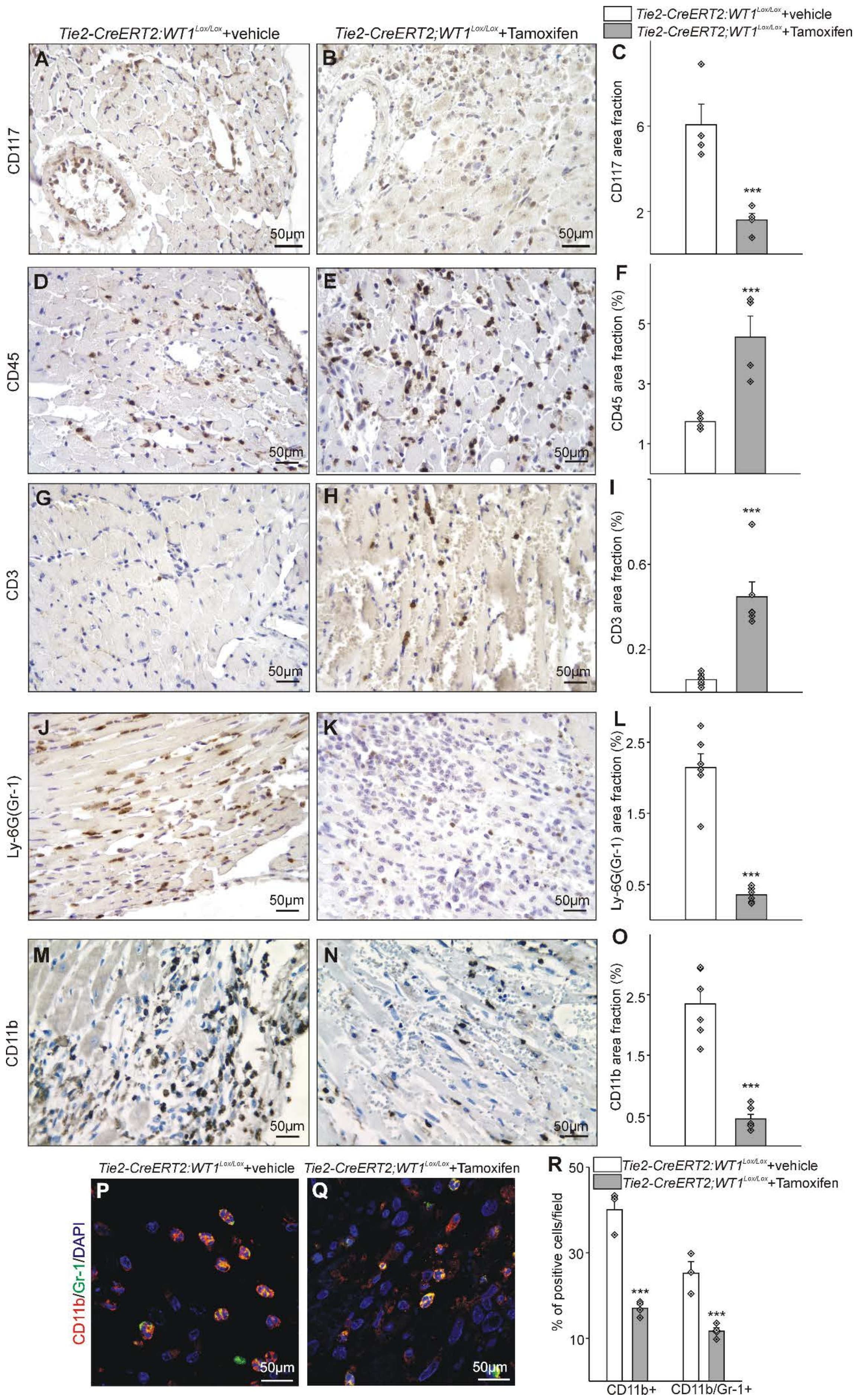

Tie2-CreERT2:WT1^Lox/Lox+vehicle
Tie2-CreERT2;WT1^Lox/Lox+Tamoxifen
A
B
C
CD117
CD117 area fraction
D
E
F
CD45
CD45 area fraction (%)
G
H
I
CD3
CD3 area fraction (%)
J
K
L
Ly-6G(Gr-1)
Ly-6G(Gr-1) area fraction (%)
M
N
O
CD11b
CD11b area fraction (%)
50μm
***
P
Q
R
CD11b/Gr-1/DAPI
% of positive cells/field
CD11b+
CD11b/Gr-1+

Figure S3: Less hematopoietic and immunosuppressive cell invasion and higher lymphocyte counts mark cardiac lesions of *Tie2-CreERT2;Wt1$^{lox/lox}$*+Tamoxifen animals in the acute phase after MI. Photomicrographs of CD117 immunostained left ventricles of (A) *Tie2-CreERT2;Wt1$^{lox/lox}$*+vehicle and (B) *Tie2-CreERT2;Wt1$^{lox/lox}$*+Tamoxifen animals. (C) Quantification of CD117 signal area fractions of left ventricle sections from *Tie2-CreERT2;Wt1$^{lox/lox}$*+vehicle controls (*n* = 4) and *Tie2-CreERT2;Wt1$^{lox/lox}$*+Tamoxifen (*n* = 6*)* animals. Photomicrographs of CD45 immunostained left ventricles of (D) *Tie2-CreERT2;Wt1$^{lox/lox}$*+vehicle and (E) *Tie2-CreERT2;Wt1$^{lox/lox}$*+Tamoxifen animals. (F) Quantification of CD45 signal area fractions of left ventricle sections from *Tie2-CreERT2;Wt1$^{lox/lox}$*+vehicle controls (*n* = 4) and *Tie2-CreERT2;Wt1$^{lox/lox}$*+Tamoxifen (*n* = 6*)* animals. Photomicrographs of CD3 immunostained left ventricles of (G) *Tie2-CreERT2;Wt1$^{lox/lox}$*+vehicle and (H) *Tie2-CreERT2;Wt1$^{lox/lox}$*+Tamoxifen animals. (I) Quantification of CD3 signal area fractions of left ventricle sections from *Tie2-CreERT2;Wt1$^{lox/lox}$*+vehicle controls (*n* = 4) and *Tie2-CreERT2;Wt1$^{lox/lox}$*+Tamoxifen (*n* = 6*)* animals. Photomicrographs of Ly-6G(Gr-1) immunostained left ventricles of (J) *Tie2-CreERT2;Wt1$^{lox/lox}$*+vehicle and (K) *Tie2-CreERT2;Wt1$^{lox/lox}$*+Tamoxifen animals. (L) Quantification of Ly-6G(Gr-1) signal area fractions of left ventricle sections from *Tie2 -CreERT2;Wt1$^{lox/lox}$*+vehicle controls (*n* = 4) and *Tie2-CreERT2;Wt1$^{lox/lox}$*+Tamoxifen (*n* = 6*)* animals. Photomicrographs of CD11b immunostained left ventricles after acute MI of (M) *Tie2-CreERT2;Wt1$^{lox/lox}$*+vehicle and (N) *Tie2-CreERT2;Wt1$^{lox/lox}$*+Tamoxifen animals. (O) Quantification of CD11b signal area fractions of left ventricle sections from *Tie2-CreERT2;Wt1$^{lox/lox}$*+vehicle controls (*n* = 4) and *Tie2-CreERT2;Wt1$^{lox/lox}$*+Tamoxifen (*n* = 6*)* animals after acute MI. Representative confocal images of CD11b (red) and Ly-6G (Gr-1) (green) double-labelling in the left-ventricular infarct zone from (P) *Tie2-CreERT2;Wt1$^{lox/lox}$*+vehicle and (Q) *Tie2-CreERT2;Wt1$^{lox/lox}$*+Tamoxifen animals. DAPI (blue) served as counterstain. (R) Quantification of left-ventricular relative cell numbers positive for CD11b alone or double positive for CD11b and Ly-6G (Gr-1) of *Tie2-CreERT2;Wt1$^{lox/lox}$*+vehicle controls (*n*=3) and *Tie2-CreERT2;Wt1$^{lox/lox}$*+Tamoxifen (*n*=4*)* animals after acute MI. Data are mean ± SEM. ***$p < 0.001$.

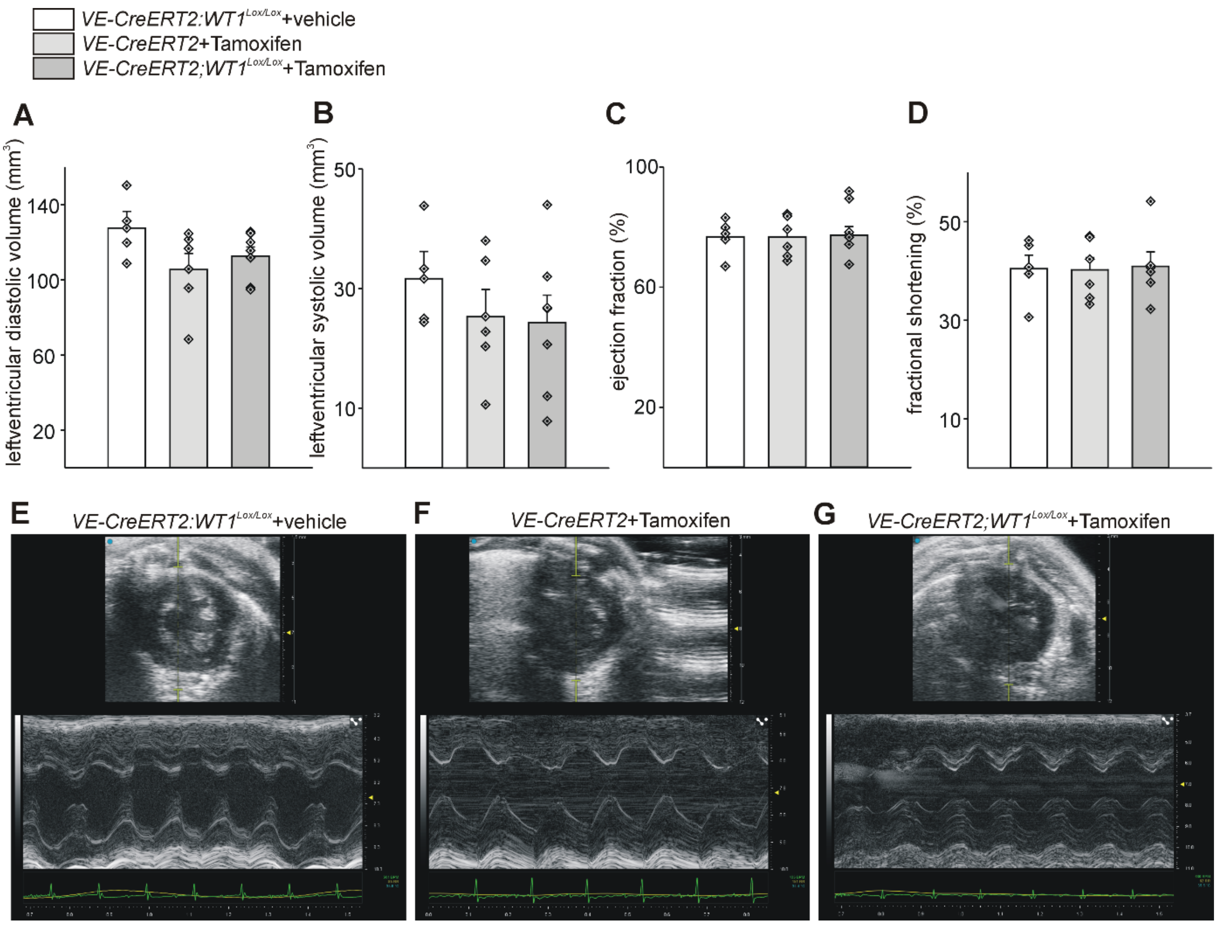

Figure S4: Baseline echocardiographic parameters of animals with restricted conditional inducible knockout of WT1 in endothelial cells without myocardial infarction. Left-ventricular diastolic volume (A), left-ventricular systolic volume (B), ejection fraction (C), and fractional shortening (D) as echocardiographic parameters of *VE-CreERT2;Wt1*$^{lox/lox}$+vehicle (*n* = 5); *VE-CreERT2*+Tamoxifen (*n = 6),* and *VE-CreERT2;Wt1*$^{lox/lox}$+Tamoxifen (*n* = 6) animals without MI. Representative echocardiographic images for *VE-CreERT2;Wt1*$^{lox/lox}$+vehicle (E), *VE-CreERT2*+Tamoxifen (F), and *VE-CreERT2;Wt1*$^{lox/lox}$+Tamoxifen (G) animals. Data are mean ± SEM.

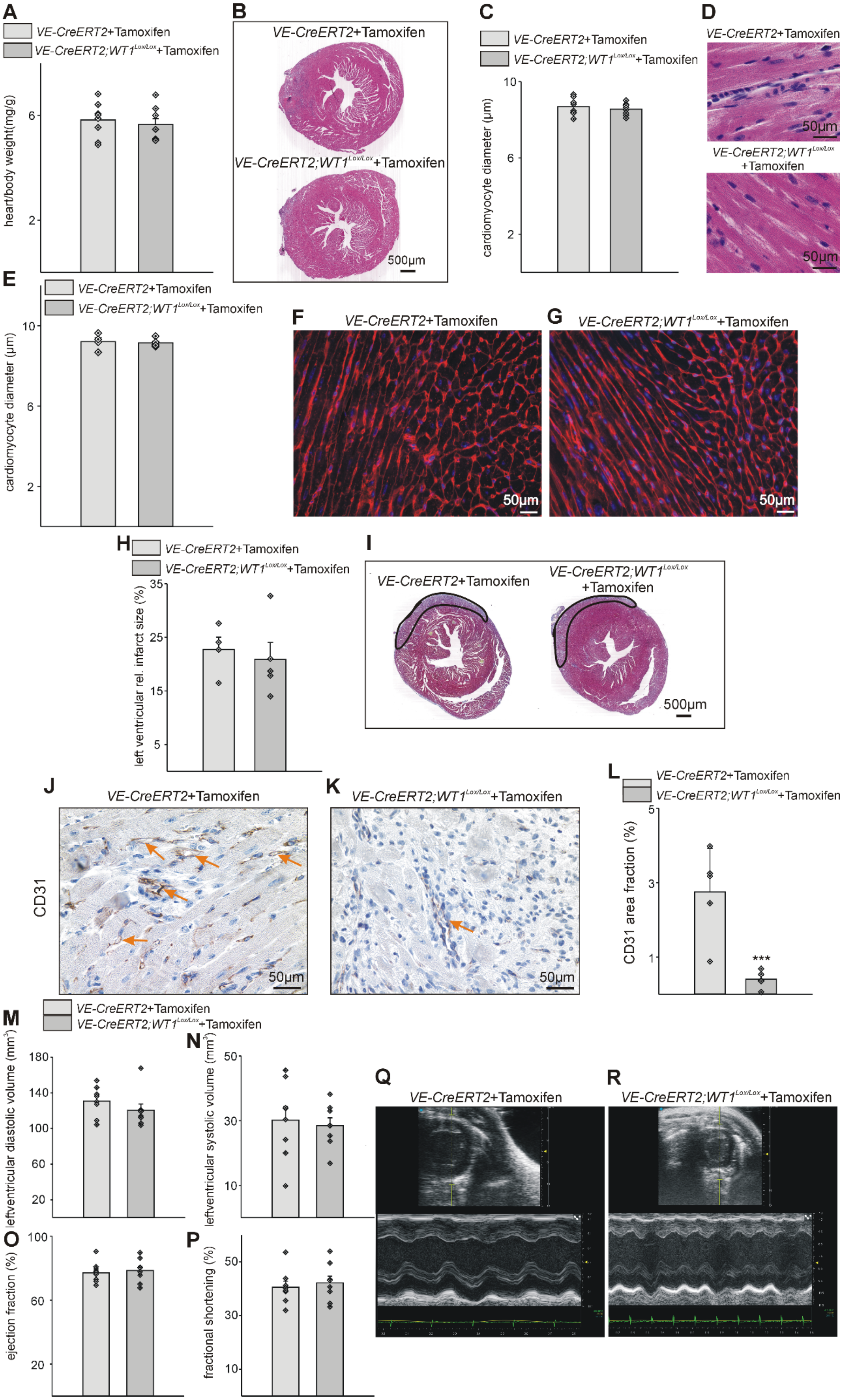
A
VE-CreERT2+Tamoxifen
VE-CreERT2;WT1Lox/Lox+Tamoxifen
heart/body weight(mg/g)
B
C
cardiomyocyte diameter (µm)
D
50µm
500µm
E
F
G
H
left ventricular rel. infarct size (%)
I
J
K
CD31
L
CD31 area fraction (%)
***
M
leftventricular diastolic volume (mm³)
N
leftventricular systolic volume (mm³)
O
ejection fraction (%)
P
fractional shortening (%)
Q
R

Figure S5: Restricted conditional inducible knockout of WT1 in endothelial cells diminishes vessel density but does not significantly influence morphological and functional cardiac parameters following MI. Respective heart-to-body weight ratios (A), photomicrographs of HE-stained heart sections (B), quantification of cardiomyocyte diameters based on measurements from HE-stained heart sections (C), and high power photomicrographs of HE-stained heart sections showing individual cardiomyocytes (D) from *VE-CreERT2*+Tamoxifen controls, (*n* = 8); and *VE-CreERT2;Wt1*$^{lox/lox}$+Tamoxifen, (*n* = 8) animals, 72 hours after MI. (E) Quantification of cardiomyocyte diameters based on measurements from WGA-stained heart sections, and high-power photomicrographs of WGA-stained heart sections showing individual cardiomyocytes (F) from *VE-CreERT2*+Tamoxifen controls (*n* = 4) and (G) *VE-CreERT2;Wt1*$^{lox/lox}$+Tamoxifen mice (*n* = 5). (H) Left ventricular infarct sizes of *VE-CreERT2*+Tamoxifen controls (*n* = 4) and *VE-CreERT2;Wt1*$^{lox/lox}$+Tamoxifen mice (*n* = 5). (I) Representative photomicrographs of Trichrome Masson-stained heart sections indicating the relative infarct size. CD31 immunostaining of heart sections from (J) *VE-CreERT2*+Tamoxifen controls (*n* = 5) and (K) *VE-CreERT2;Wt1*$^{lox/lox}$+Tamoxifen animals (*n* = 5) after MI. Orange arrows indicate some of the visible vessels in the respective photomicrographs. (L) Quantification of CD31 signal area fraction (*VE-CreERT2*+Tamoxifen controls (*n* = 5) and *VE-CreERT2;Wt1*$^{lox/lox}$+Tamoxifen animals (*n* = 5). Left-ventricular diastolic volume (M), left-ventricular systolic volume (N), ejection fraction (O), and fractional shortening (P) as echocardiographic parameters of *VE-CreERT2;Wt1*$^{lox/lox}$+Tamoxifen, *(n* = 8); *VE-CreERT2;Wt1*$^{lox/lox}$+vehicle, (*n = 8)*; *VE-CreERT2*+Tamoxifen, (*n = 8)* animals 72hrs after MI. Representative echocardiographic images for *VE-CreERT2*+Tamoxifen (Q), and *VE-CreERT2;Wt1*$^{lox/lox}$+Tamoxifen (R) animals after MI. Data are mean ± SEM. ***$p < 0.001$.

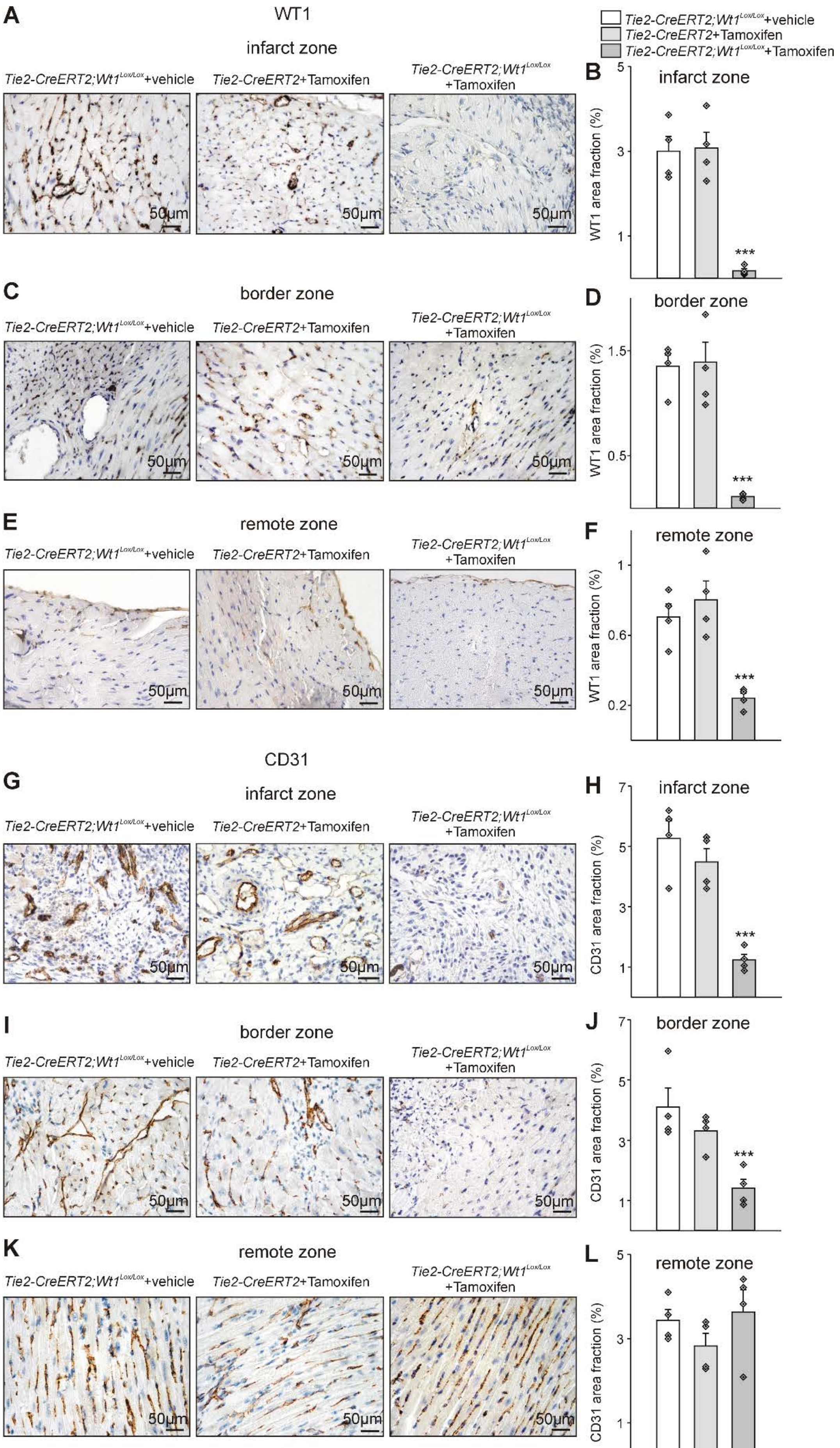

A
WT1
infarct zone
Tie2-CreERT2;Wt1Lox/Lox+vehicle
Tie2-CreERT2+Tamoxifen
Tie2-CreERT2;Wt1Lox/Lox+Tamoxifen
50μm
B
infarct zone
WT1 area fraction (%)
***
C
border zone
D
border zone
E
remote zone
F
remote zone
CD31
G
infarct zone
H
infarct zone
CD31 area fraction (%)
I
border zone
J
border zone
K
remote zone
L
remote zone

Figure S6: Tie2-CreERT2-mediated WT1 loss impairs cardiac revascularization in the repair process following MI. Photomicrographs of WT1 immunostained left ventricles 3 weeks after MI in the infarct, border, and remote zone (A,C,E) of *Tie2-CreERT2;Wt1*$^{lox/lox}$+vehicle, *Tie2-CreERT2* +Tamoxifen and of *Tie2-CreERT2;Wt1*$^{lox/lox}$+Tamoxifen animals. Quantification of WT1 signal area fractions in the infarct (B), border (D), and remote (F) zone of hearts from *Tie2-CreERT2;Wt1*$^{lox/lox}$+vehicle controls (n = 4), *Tie2-CreERT2*+Tamoxifen (n = 4), and *Tie2-CreERT2;Wt1*$^{lox/lox}$+Tamoxifen (n = 4) animals 3 weeks after MI. Photomicrographs of CD31 immunostained left ventricles 3 weeks after MI in the infarct, border, and remote zone (G, I, K) of *Tie2-CreERT2;Wt1*$^{lox/lox}$+vehicle, *Tie2-CreERT2* +Tamoxifen and of *Tie2-CreERT2;Wt1*$^{lox/lox}$+Tamoxifen animals. Quantification of CD31 signal area fractions in the infarct (H), border (J), and remote (L) zone of hearts from *Tie2-CreERT2;Wt1*$^{lox/lox}$+vehicle controls (n = 4), *Tie2-CreERT2*+Tamoxifen (n = 4), and *Tie2-CreERT2;Wt1*$^{lox/lox}$+Tamoxifen (n = 4) animals 3 weeks after MI. Data are mean ± SEM. ***p < 0.001.

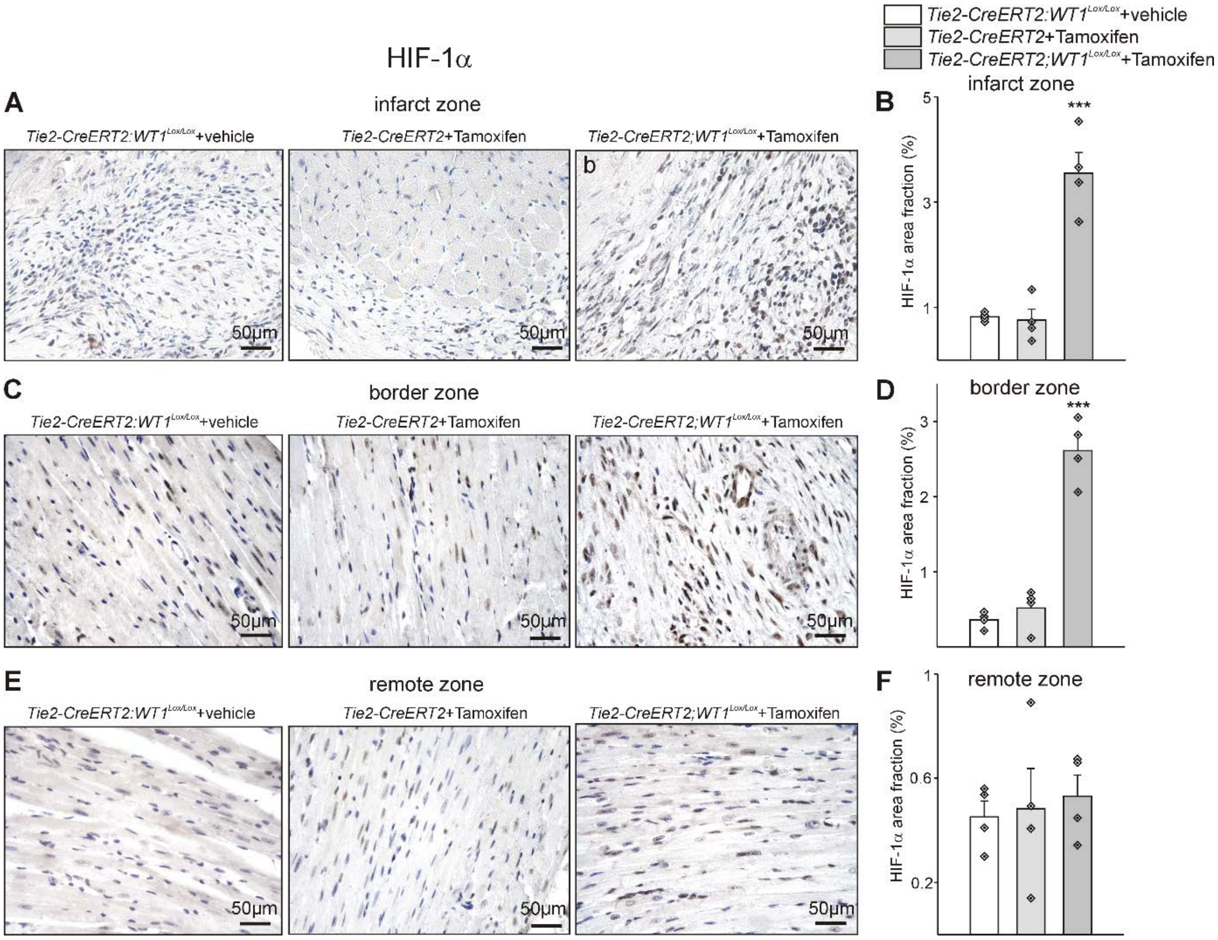

Figure S7: High levels of HIF-1α protein correlate to diminished vascularization in cardiac repair due to loss of WT1. Photomicrographs of HIF-1α immunostained left ventricles 3 weeks after MI in the infarct, border, and remote zone (A, C, E) of *Tie2-CreERT2;Wt1*$^{lox/lox}$+vehicle, *Tie2-CreERT2* +Tamoxifen and of *Tie2-CreERT2;Wt1*$^{lox/lox}$+Tamoxifen animals. Quantification of HIF-1α signal area fractions in the infarct (B), border (D) and remote (F) zone of hearts from *Tie2 -CreERT2;Wt1*$^{lox/lox}$+vehicle controls (n = 4), *Tie2-CreERT2*+Tamoxifen (n = 4), and *Tie2-CreERT2;Wt1*$^{lox/lox}$+Tamoxifen (n = 4) animals 3 weeks after MI. Data are mean ± SEM. ***$p < 0.001$.

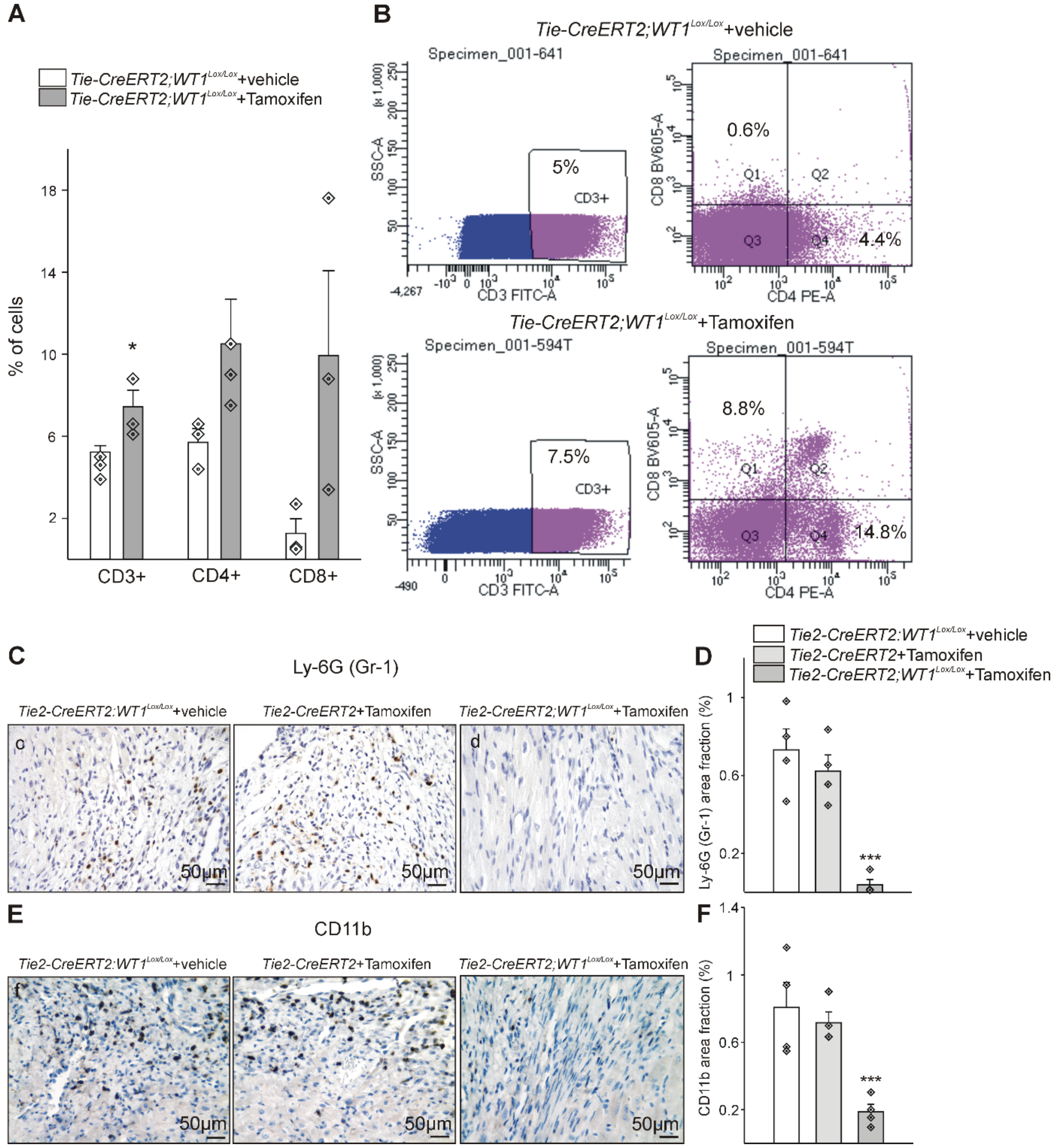

Figure S8: Higher lymphocyte counts and decreased numbers of immunosuppressive cells in *Tie2-CreERT2;Wt1*$^{lox/lox}$+Tamoxifen hearts after MI. (A) Quantification of FACS analysis of the fraction of CD3$^+$, CD3$^+$CD4$^+$, and CD3$^+$CD8$^+$lymphocytes from hearts of *Tie2-CreERT2;Wt1*$^{lox/lox}$+vehicle controls (*n*=3) and *Tie2-CreERT2;Wt1*$^{lox/lox}$+Tamoxifen (*n* = 3*)* animals 3 weeks after MI. Representative flow cytometry data (B) of a *Tie2-CreERT2;Wt1*$^{lox/lox}$+vehicle control heart (upper panel) and a *Tie2-CreERT2;Wt1*$^{lox/lox}$+Tamoxifen heart (lower panel) for lymphocyte quantification. Photomicrographs of Ly-6G (Gr-1) immunostained left ventricles 3 weeks after MI (C) of *Tie2-CreERT2;Wt1*$^{lox/lox}$+vehicle, *Tie2-CreERT2* +Tamoxifen, and *Tie2-CreERT2;Wt1*$^{lox/lox}$+Tamoxifen animals. (D) Quantification of Ly-6G(Gr-1) signal area fractions of left ventricle sections from *Tie2-CreERT2;Wt1*$^{lox/lox}$+vehicle controls (*n* = 4), *Tie2-CreERT2*+Tamoxifen (*n* = 4), and *Tie2-CreERT2;Wt1*$^{lox/lox}$+Tamoxifen (*n* = 4*)* animals. Photomicrographs of CD11b immunostained left ventricles 3 weeks after MI (E) of *Tie2-*

CreERT2;Wt1$^{lox/lox}$+vehicle, *Tie2-CreERT2* +Tamoxifen, and *Tie2-CreERT2;Wt1*$^{lox/lox}$+Tamoxifen animals. (F) Quantification of CD11b signal area fractions of left ventricle sections from *Tie2* -*CreERT2;Wt1*$^{lox/lox}$+vehicle controls (*n* = 4), *Tie2-CreERT2*+Tamoxifen (*n* = 4), and *Tie2-CreERT2;Wt1*$^{lox/lox}$+Tamoxifen (*n* = 4*)* animals 3 weeks after MI. Data are mean ± SEM. *p < 0.05; ****p* < 0.001.

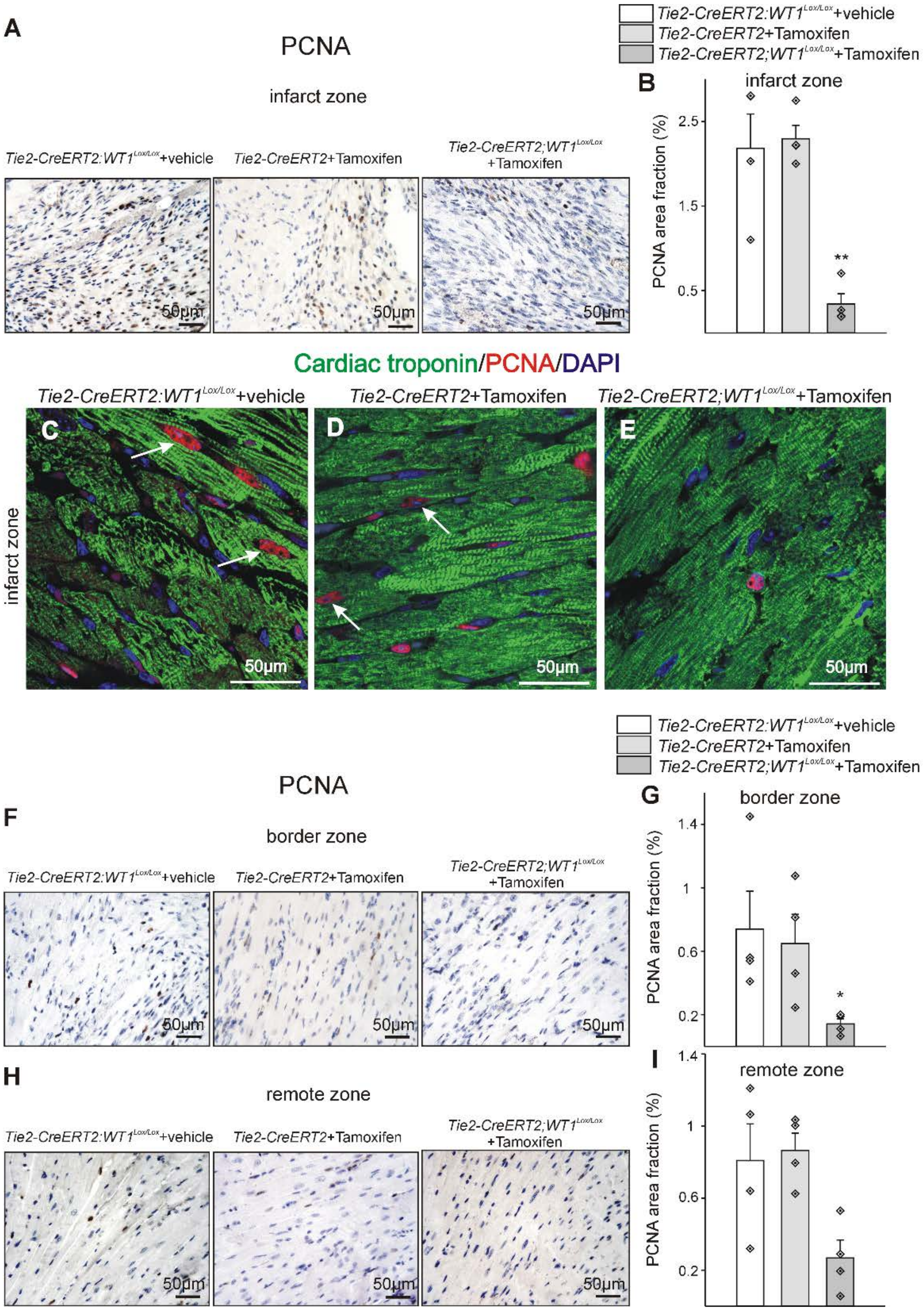

Figure S9: Diminished proliferation in *Tie2-CreERT2;Wt1*$^{lox/lox}$+Tamoxifen hearts after MI. Photomicrographs of PCNA immunostained left ventricles 3 weeks after MI in the infarct, border, and remote zone (A, F, H) of *Tie2-CreERT2;Wt1*$^{lox/lox}$+vehicle, *Tie2-CreERT2* +Tamoxifen, and *Tie2-CreERT2;Wt1*$^{lox/lox}$+Tamoxifen animals. Quantification of PCNA signal area fractions in the infarct (B), border (G), and remote (I) zone of hearts from *Tie2-CreERT2;Wt1*$^{lox/lox}$+vehicle controls (n = 4), *Tie2-CreERT2*+Tamoxifen (n = 4), and *Tie2-CreERT2;Wt1*$^{lox/lox}$+Tamoxifen (n = 4) animals 3 weeks after MI. Representative confocal images of cardiac troponin (green) and PCNA (red) double-labelling of the infarct zone from heart sections from *Tie2-CreERT2;Wt1*$^{lox/lox}$+vehicle (C), *Tie2-CreERT2*+Tamoxifen (D), and *Tie2-CreERT2;Wt1*$^{lox/lox}$+Tamoxifen (E) animals. DAPI (blue) served as nuclear counterstain. White arrows indicate PCNA positive cardiomyocytes. Data are mean ± SEM. ***$p < 0.001$.

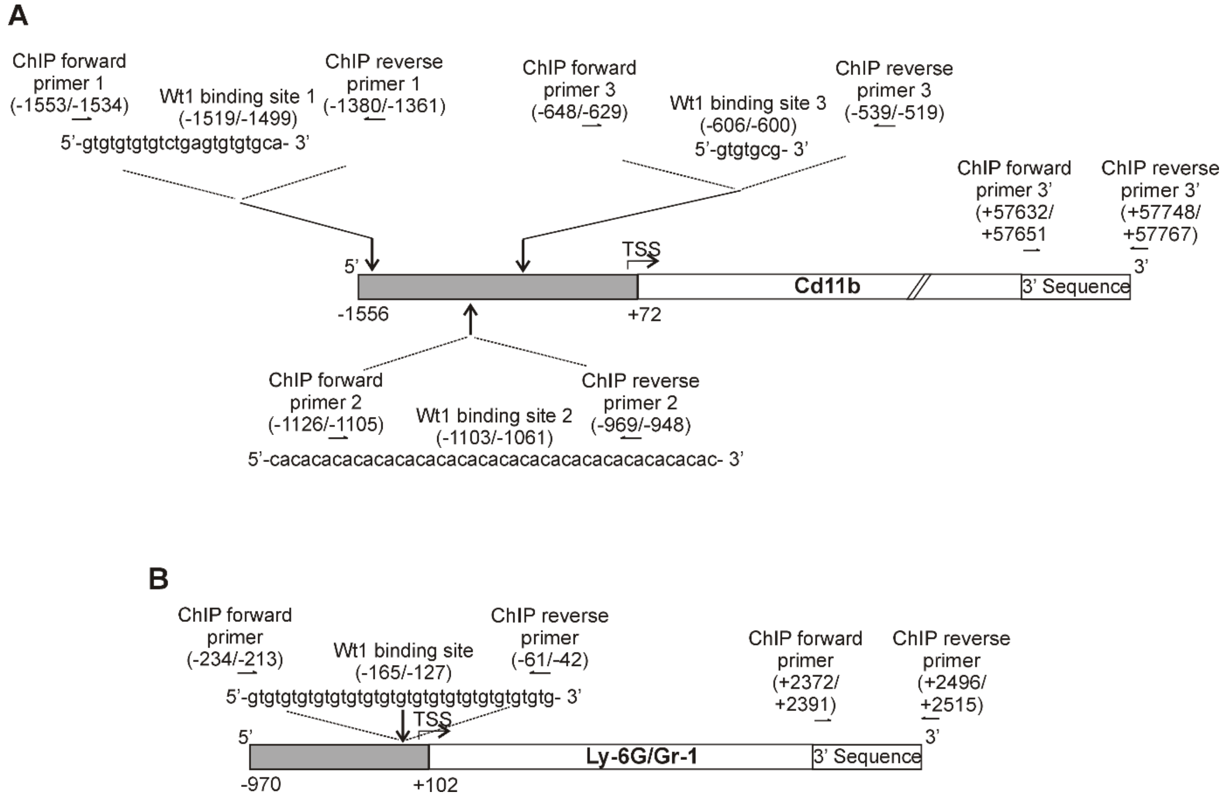

Figure S10: Schematic representation of the putative WT1-binding sites and indication of the CHIP oligonucleotide positions in the CD11b (A) and Ly-6G(Gr-1) (B) promoters.
